# Validity of pulse oximetry measures for heart rate and oxygen saturation during profound hypoxia in normobaric simulated extreme altitudes

**DOI:** 10.1371/journal.pone.0326674

**Published:** 2025-06-23

**Authors:** Harald Vikne, Jon Arild Kjeserud, Willy Westgaard, Ruben Baalsrud Westlie, Jan Ivar Kåsin, Jon Ingulf Medbø, Terje Gjøvaag, Nils Henrik Holmedahl

**Affiliations:** 1 Norwegian Institute of Aviation Medicine, Norwegian Armed Forces Joint Medical Services, Oslo, Norway; 2 Department of Interdisciplinary Health Sciences, Institute of Health and Society, University of Oslo, Oslo, Norway; 3 Faculty of Teacher Education, Culture and Sports, Western Norway University of Applied Sciences, Sogndal, Norway; 4 Department of Rehabilitation Science and Health Technology, Faculty of Health Sciences, Oslo Metropolitan University, Oslo, Norway; Polytechnic University of Marche: Universita Politecnica delle Marche, ITALY

## Abstract

**Introduction:**

Commercial pulse oximeters may not be well calibrated for oxygen saturations below 70%, conditions that may be met in high altitude aviation and mountaineering. We therefore examined the bias and variability of heart rate (HR) and blood oxygen saturation (SpO_2_) of four different pulse oximeters (PO) at arterial blood oxygen saturation (SaO_2_) between 55 and 100%.

**Methods:**

Seventeen healthy participants (age 33 ± 11 (mean ± standard deviation (SD)) yr) were exposed to controlled desaturation at rest by stepwise reduction of the oxygen fraction in the breathing air between 20.9 and 8%. Parallel measurements of HR (*n* = 383) and blood oxygen saturation (*n* = 304) from four pulse oximeters (RAD-97, PM100N, M7500 and Nell1-SR) and from reference instruments (by hemoximetry and electrocardiography (ECG)) were taken during the exposure. The validity was assessed in intervals of 55–70%, 70–85% and 85–100% SaO_2_ using the Bland-Altman method (bias and 95% limits of agreement (LoA)) and the root mean square error for variability. The demarcation criteria for agreement between methods were ±6 percentage points O_2_Hb saturation and ±6 bpm HR.

**Results:**

At the 85–100% SaO_2_ interval, all POs but the Nell1-SR were in agreement with the reference for O_2_ saturation. Only M7500 agreed with the reference for the 70–85% interval and none of the POs were in agreement with the reference for the 55–70% interval. The pulse oximeters and ECG were not in agreement for HR at neither the 55–70% nor the 85–100% interval but agreed at the 70–85% interval except the RAD-97. All pulse oximeters increased the bias or the variability for SpO_2_ significantly by reductions in oxygen saturation, while no systematic differences were found for HR.

**Conclusion:**

The study shows that medically approved pulse oximeters are not in agreement with reference measurements of neither blood oxygen saturation nor HR at SaO_2_ levels below 70%, and their readings should therefore be interpreted cautiously during severe desaturation.

## Introduction

Oxygen is vital for the normal physiological functions of the human body, including cellular respiration and energy production. Generalized acute hypoxia – reduced oxygen availability at the whole-body level – is therefore a health- and incapacitation risk factor when otherwise healthy persons ascend in the atmosphere such as during mountaineering or aviation and this is known as altitude or hypobaric hypoxia. Severe acute hypoxia will generally lead to a temporary decrease in cognitive speed and function [1,2], may lead to loss of consciousness [3] and origin life-threatening situations. The atmospheric O_2_ pressure decreases almost exponentially by increasing altitude [4], and without supplemental oxygen, hypobaric hypoxia in healthy individuals is a normal consequence of the reduced environmental oxygen pressure and availability at increasing altitude [5]. In addition to symptoms, generalized hypoxia may be revealed by reductions in the arterial blood oxygen saturation – that is the relative amount of oxyhemoglobin to unbound hemoglobin, using direct measurement in arterial blood by hemoximetry (SaO_2_). Although hemoximetry yields highly precise measurements of an individual’s hematological status, the procedure necessitates arterial puncture, rendering it invasive and painful, with associated risks of tissue damage and infection [6]. Moreover, the requisite sampling procedures and equipment leaving continuous monitoring of SaO_2_ challenging and less practical outside specialized facilities.

A non-invasive alternative to the measurement of SaO_2_, pulse oximetry, was invented early in the 1970s [7] and is a photoplethysmographic (PPG) method for indirect, continuous measurement of arterial blood oxygen saturation (SpO_2_) and heart rate (HR) through the repetitive arterial blood pulses [8]. Pulse oximetry uses light of different wavelengths to illuminate blood as a method to indirectly estimate the percentage of oxygenated versus deoxygenated hemoglobin in real-time. Oxygen saturation is estimated by measuring the change in the ratio of red to infrared light absorbed by oxyhemoglobin and deoxygenated hemoglobin and received by a photodetector throughout a blood pulse, based on the assumption that the venous saturation component does not change. The ratio of absorbed red to infrared light is compared with an internal standard curve for oxygen saturation, based on empirical data of the specific pulse oximeter manufacturer [9,10]. Pulse oximetry has gained a wide range of uses from hospital care to altitude, exercise and sports science [11–14] due to its non-invasive nature, ease of use, and reasonably robust measurements. Manufacturers of pulse oximeters continuously improve the hardware, algorithms and performance of their instruments that are validated according to ISO standards for medical approvement of the manufacturer or a specialized test laboratory. The measures of SpO_2_ and HR of the pulse oximeters and sensors are validated at an SaO_2_ interval between 70 and 100% by using hemoximetry and electrocardiography (ECG) as reference standards, respectively. The accuracy and precision are assessed by use of Bland & Altman’s bias (mean difference between the reference and the pulse oximeter) and 95% limits of Agreement (1.96 times the SD of the bias) and the root mean square error of the difference (RMSE). Typically, medically approved pulse oximeters and sensors report an overall RMSE of ≈ 2–3 percentage points (pp) or a corresponding 95% limit of agreement (LoA) of 4–6% across the 70–100% saturation interval when compared with a reference [15–17]. There are, however, only few independent comparative examinations of the performance of pulse oximeters [18].

Pulse oximetry is generally subject to various threats to validity [9,10], including skin color [19], use of nail polish [20], motion [18,21], perfusion at the assessment site [21,22], probe types and sites of measurement [23,24], and fractions of the dyshemoglobins COHb and MetHb [25–27]. All these factors can affect the accuracy and precision of pulse oximeter performance. Differences between the pulse oximeter manufacturers in hardware, software and algorithms may also introduce variation in measurement errors. Previous examinations have revealed that the uncertainty of measurement of pulse oximeters generally increases by decreasing oxygen saturation [18,24,28,29]. While the accuracy and precision of pulse oximeters typically are high in humans (i.e., display minor bias, LoAs, and RMSE values) at high saturation intervals (90–100% SaO_2_), the variability is increased in the intervals of 70–80% saturation [30]. This increased variability by desaturation seems to be a general finding for pulse oximetry and is also a challenge in other mammals as well [31,32]. In several situations humans may experience Hb oxygen saturation below the limits used for medical approval of pulse oximeters. For example, may persons with severe obstructive sleep apnea drop towards the 70% O_2_ saturation validation limits at a moderate altitude of 2600 m [33]. During exposures in environmental conditions that may be physiologically challenging, such as during extreme altitude mountaineering and during acute and prolonged simulated altitudes, healthy individuals may experience arterial oxygen saturations towards and well below 70% [34–37]. At acute decompression to about 7600 m (25 000 ft) of simulated altitude in hypobaric chambers during hypoxia recognition training (HRT), some participants may experience profound hypoxia within 3–4 min, and the concurrent arterial oxygen saturation may even fall below 60% SpO_2_ [34,38]. There is consequently a gap between the range of measurement used in standard experiments for medical approbation and those that may be achieved during exposures to decreased atmospheric pressure. At saturation levels below 70%, the hemoglobin–oxygen dissociation curve is at its steepest [39]. If measurement variability increases even further below 70% saturation, there is a significant risk of failing to detect extreme desaturation, potentially leading to severe health deterioration and incapacitation.

The overall purpose of this study was to investigate and compare the validity of four medically approved commercial finger pulse oximeters on oxygen saturation and heart rate between 55 and 70% SaO_2_ using reference measurement of hemoximetry and electrocardiogram in healthy participants. Here we provide empirical data on the validity of four pulse oximeters at arterial oxygen saturation less than 70%, and the results of the study may therefore be useful for researchers, healthcare professionals in the treatment of people with very low oxygen saturation, mountaineers and in hypoxia recognition training of aircrews.

## Methods

### Approach to the problem

**Fig 1:**
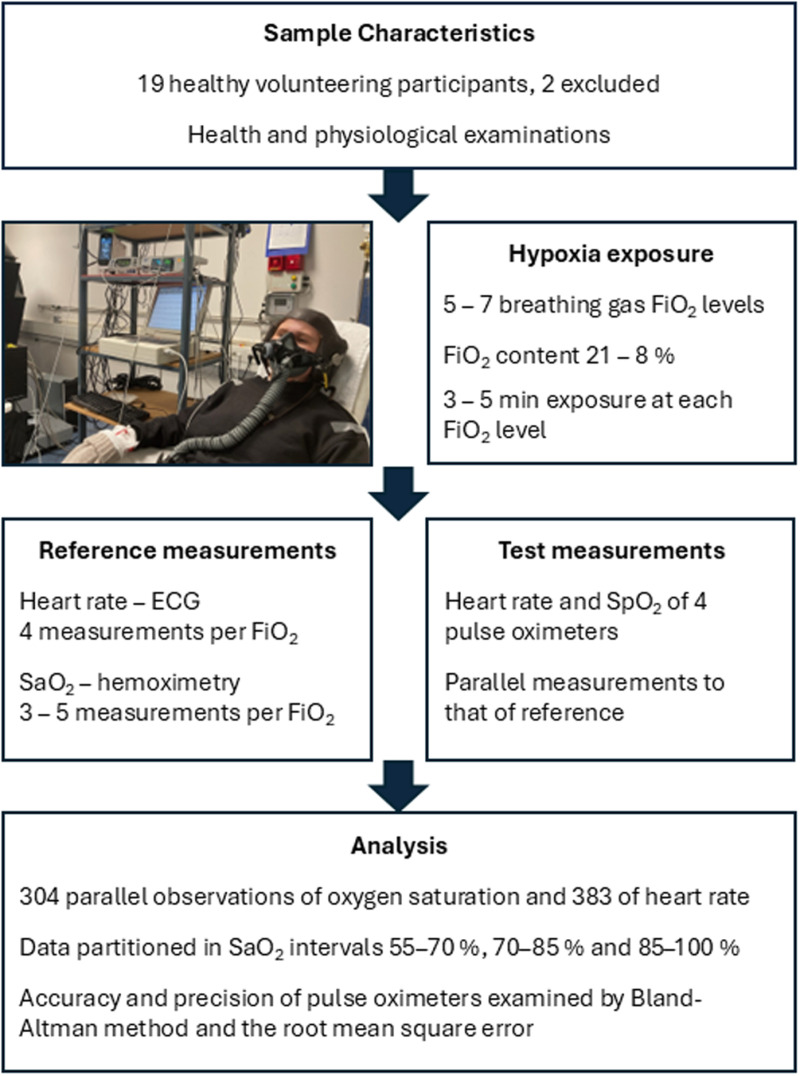
Overview of the study.

To examine the validity of four different pulse oximeters at severe hypoxic conditions, 17 healthy participants were stepwise desaturated using reduced fractions of inspired O_2_ towards 55% O_2_ saturation, and parallel measurements of pulse oximetry and reference instruments (hemoximeter and electrocardiography) were taken. Four finger pulse oximeters were examined simultaneously on each participant. The participants were exposed to normobaric hypoxia using breathing air mixtures containing reduced oxygen concentration supplied by a gas blender. Five to seven different air mixtures were given in a stepwise manner to introduce stable oxygen saturation plateaus between 55 and 100%. At each oxygen saturation plateau, 3–5 simultaneous measurements from the reference apparatuses and the pulse oximeters were taken, separated by ~30 s. To examine and compare the validity of the pulse oximeters below and above the 70% saturation demarcation level, the data were partitioned in three equally wide saturation intervals of 15 percentage points (pp), consisting of one interval below 70% (55–70%) and two intervals above (70.1–85% and 85.1–100%). The validity was examined within each saturation interval using the Bland & Altman’s limits of agreement method [40] adjusted for repeated measurements [41,42], and further by the root mean square error (RMSE) and then compared to the others. See Fig 1 for overview of the study. The study followed the ethical guidelines set by the Declaration of Helsinki, and pre-approval was given by the Regional Committee for Medical and Health Research Ethics, region South-East A (ID NO. 229473). The General Data Protection Regulation (GDPR) was followed, and the local Data Protection Authority was consulted a priori. All volunteers gave their informed written consent prospectively. The study protocol was a priory registered at the Open Science Framework Registries (Registration DOI: https://doi.org/10.17605/OSF.IO/UDX7G).

### Participants: Recruitment, health examination and anthropometric information

Nineteen healthy adult women (*n* = 8) and men (*n* = 11) from the greater Oslo area volunteered to participate after promoting the study through flyers and information meetings at local aero clubs and universities. The data collection period lasted from the 25^th^ of November 2022 to the 10^th^ of March 2023. All participants received oral and written information about the study, about possible risks and disadvantages in participation. Potential participants were initially examined by a medical doctor, and descriptive variables were measured. Persons suffering from dizziness or fainting tendencies, anemia, circulatory or respiratory disorders or from kidney or liver disease, were excluded. Persons using anticoagulants, being allergic to lidocaine, prilocaine or other similar local anesthetics, had bleeding or coagulation disorders, were pregnant or breastfeeding, obese (BMI > 29.9 kg/m^2^), or using Continuous Positive Air Pressure (CPAP), feared injections (belonephobia/hemophobia/algophobia) or were smoking, were also excluded. The participants had to display normal levels of hemoglobin (men: > 13.4 g/100 ml, women > 11.7 g/100 ml), carboxyhemoglobin (COHb, < 3%), methemoglobin (MetHb, ≤ 1.1%) and speak Norwegian fluently. Two participants were excluded due to low Hb concentration or unsuccessful arterial line insertion. A total of 17 participants (7 women, 10 men; age range 20–54 years) were thus accepted as subjects, and all completed the exposure intervention. The anthropometric data are reported in Table 1. The sample size was calculated a priori in SciStat using the Lu et al. [43] method for Bland-Altman analyzes. Type I and type II errors were set to 5% and 20%, respectively. All input values were taken from the reported data of the reference monitor at the lowest oxygen saturation interval 68.5–80% (Table 2) in the recent study of Louie et al. 2018 [18]. The expected mean difference between the measurement methods (bias) was 0.9 pp (percentage points), and the expected standard deviation (SD) of the difference was 2.1 pp. The maximum allowable difference between the methods was set to 5.3 pp, slightly higher than the LoA, including the 95% CI reported by Louie et al. [18], to account for the fact that the planned saturation would be lower than that in the study of Louie et al. [18]. This resulted in a minimum sample size of 96 when taking into account a dropout rate of 25%. We thus needed about 20 participants with five observations per participant to be able to determine whether the measurements of the pulse oximeter and the reference methods are similar.

**Table 1:** Anthropometrics, body composition and physical activity patterns of the participants in the study (*n* = 17).

Descriptive variable	Value
**Women/men (number)**	7/10
**Age (years)**	33.2 (11.1)
**Body height (cm)**	176.1 (10)
**Body weight (kg)**	80.1 (11.6)
**Body mass index (kg/cm**^**2**^)	25.8 (2.7)
**Fat-free mass (kg)**	61.5 (10.5)
**Muscle mass (kg)**	34.8 (6.3)
**Muscle mass (%)**	43.4 (4)
**Body fat mass (kg)**	18.6 (5.9)
**Body fat mass (%)**	23.2 (6.4)
**Physical activity (MET-min/week)** ^*^	2339.5 (1479.5)
**Physical activity (categorical score: *n* – %)**	High: 10–58.8% Moderate: 6–35.3% Low: 1–5.9%

Data are number and mean (± standard deviations) of absolute and relative values.

* International Physical Activity Questionnaire, short form (IPAQ SF) score is reported as median value and interquartile range as proposed by the IPAQ Research committee.

**Table 2:** Hematological data of all participants at baseline (*n* = 17).

Descriptive variable	Mean (SD)
**THb (g/100 ml)**	14.6 (1.4)
**HCT (%)**	44.9 (4.1)
**FO**_**2**_ **Hb (%)**	96.0 (0.6)
**FCOHb (%)**	1.1 (0.2)
**FHHB (%)**	2.1 (0.5)
**FMetHb (%)**	0.8 (0.1)
**pH (-log10[H+])**	7.37 (0.05)
***p*CO**_**2**_ **(kPa)**	5.0 (0.5)
***p*O**_**2**_ **(kPa)**	12.9 (0.9)
***p***_**50**_ **(kPa)**	3.59 (0.2)
**HCO**_**3**_^**–**^ **(mMol/L)**	21.0 (2.1)

Data are the mean (SD) of three measurements per participant.

Abbreviations. THB, total hemoglobin; HCT, hematocrit; FO_2_Hb, fraction of oxyhemoglobin; FCOHb, fraction of carboxyhemoglobin; FHHb, fraction of deoxyhemoglobin; FMetHb, fraction of methhemoglobin; *p*CO_2_, pressure of carbondioxide; *p*O_2_, pressure of oxygen; *p*_50_, pressure where hemoglobin is 50% saturated; HCO_3_^–^, bicarbonate; SD is standard deviation.

All potential participants were examined prior to the exposure by a medical doctor, who also was present during the intervention. Pulmonary function was assessed by measuring forced vital capacity (FVC, l), forced expiratory flow first second (FEV_1_, l) and peak expiratory flow (PEF, L s^-1^) (Spirare 3 spirometry, Diagnostica AS, Norway). The best of three consecutive measurements was used further. Body composition (absolute (kg) and relative (%) fat and muscle mass and fat-free mass (kg)) was measured once by the impedance method (InBody 720, InBody USA, CA, USA). To estimate body compositions, we used the internal software standard algorithms and the standardized procedures recommended by the manufacturer.

Resting 12-lead electrocardiography was measured once in the supine position (Spirare 3 ECG, Diagnostica AS, Norway). Systolic (SP) and diastolic (DP) blood pressure (mmHg) was measured thrice in the sitting position (M7 Intelli IT, Omron Healthcare Co., Japan), and the mean of the measurements was used. Mean arterial pressure (MAP) was calculated as MAP = DP + 1/3(SP – DP). Self-reported physical activity behavior (MET-minutes/week) was measured using the Norwegian version of the International Physical Activity Questionnaire, short form (IPAQ SF) [44] conducted as an interview. Hematological variables (total hemoglobin (g/100 ml), hematocrit (%), O_2_Hb (%), SaO_2_ (%), COHb (%), HHB (%), MetHb (%), pH, *p*CO_2_ (kPa), *p*O_2_ (kPa), *p*_50_ (kPa) and HCO_3_^–^ (mmol/L)) were measured using ABL820 FLEX (Radiometer Medical ApS, Denmark)), and the mean of three samples were used further. Resting hematological data are displayed in Table 2 and hemodynamics and pulmonary function in Table 3.

**Table 3:** Hemodynamics and pulmonary function of the participants (*n* = 17).

Descriptive variable	Mean (SD)	% of predicted
**Heart rate (bpm)**	62.2 (10.6)	
**Systolic blood pressure (mmHg)**	125.2 (10.2)	
**Diastolic blood pressure (mmHg)**	81.2 (7.5)	
**Mean arterial pressure (mmHg)**	95.9 (7.2)	
**Forced vital capacity (L)**	4.9 (0.8)	103.9 (13.4)
**Forced expiratory volume in 1 s (L)**	3.9 (0.7)	101.3 (16.7)
**Peak expiatory flow (L s**^**-1**^)	9.7 (1.7)	114.7 (19.1)
**Anatomic pulmonary dead space (mL)**	154.7 (25.0)	

All data are given as mean (SD).

### Instrumentation

#### Pulse oximeter monitors and sensors.

Three factory new standalone, commercially available pulse oximeter monitors from three different manufacturers, RAD-97 (Masimo Corp., CA, USA), PM100N (Nellcor, Medtronic, MN, USA), Model 7500 (Nonin Medical, Inc., MN, USA) and in addition a Nell1-SR printed circuit board assembly (Nellcor, Medtronic, MN, US) within a gas mixer (ROBD2, see description below) were examined. The three finger transmittance sensors used by their respective standalone pulse oximeters were of a soft holster type (Masimo LNCS-DBI, Nellcor Flexmax and Nonin 8000SM), and the sensor for the Nell1-SR was a Nellcor Oximax compatible pinch type (S410-70P0, Cables & Sensors, FL, USA), which was the standard sensor setup for the ROBD2 gas blender. All sensors were new and obtained to the present study. The four finger sensors were randomly assigned to the second and fourth fingers of both hands for each participant using the RAND() function in Excel. The skin at the measuring areas was cleansed with 70% isopropanol wipes, and any nail polish was removed. The monitor sample averaging time intervals were adjusted to comparable levels for all pulse oximeters (RAD-97 (2–4 s averaging and FastSat algorithm weighing based on signal quality), PM100N (2–4 s), Model 7500 (PureSat averaging, using 4 beat exponential moving averaging 3 s) and the Nell1-SR (2–3 s). All pulse oximeters had a resolution of 1 pp for SpO_2_ in the measurement range 1–100% and further 1 beat per minute (bpm) for heart rate (Model 7500 (in the range 18–200 bpm), PM100N (20–250 bpm), RAD-97 (0–240 bpm). The updating frequency was reported to be 1 Hz for RAD-97, PM100N and Model 7500. For Nell1-SR, it was measured to 1 Hz using LabView software (National Instruments, TX, USA). Specified for the adult population and during non-motion conditions, all pulse oximeter monitor manufacturers reported the measurement accuracy of SpO_2_ to be 2% RMSE in the 70–100% measurement range. For HR the reported accuracy was 3 bpm RMSE for all monitors specified for the 25–240 bpm measurement range for RAD-97, 20–250 bpm for PM100N, 18–300 bpm for Model 7500 and 20–250 bpm for Nell1-SR. It is noted by the manufacturers that accuracy also varies by sensor type. Data from the pulse oximeters were sampled continuously during the intervention and transferred to a standalone PC for synchronization using LabView (see below for details).

#### Reference standards.

Arterial blood gas measurement by hemoximetry (ABL820 FLEX, Radiometer Medical ApS, Denmark) was used as the reference measure of functional arterial oxygen saturation (SaO_2_ (O_2_Hb/[Hb + O_2_Hb])). The hemoximeter was automatically calibrated, and quality control measurements were performed before and after experiments according to the manufacturer’s instructions. The hemoximeter had a measurement resolution of 0.1% pp for SaO_2_. An indwelling, 20-gauge artery catheter was placed in the radial artery of each participant after local anesthesia injections (Xylocain, 20 mg/ml) given in the skin to limit discomfort with the procedure. We mainly used the left hand of the participants, but for a few participants the right hand was used. Three to five blood samples (1.5 ml each) were taken at baseline (20.9% O_2_) and per reduced oxygen content gas (*n* = 4–7) for a total of 15–21 samples per participant. For most participants the blood sampling took about 3–5 s to complete, but for some participants the sampling took up to 15 s to obtain the necessary volume. The volume blood sampled was mixed thoroughly before analysis to ensure uniformity. Since blood collection takes a few seconds, the measured values are considered to represent an average over the sampling period. A 12-lead electrocardiography (CardioLaptop AT-110, Schiller AG, Switzerland) was used as a reference measure of heart rate (HR) and for health monitoring of the participants during the intervention. The electrodes were placed according to the manufacturer’s recommendations during the health examination and used further during the intervention. The ECG was continuously sampled at 1000 Hz, using a 50 Hz adaptive line frequency filter to suppress line interference. The sampled data were simultaneously transferred to a standalone PC at 20 Hz using LabView with a resolution of 1 bpm for synchronization. Beat for beat mean heart rate was subsequently calculated in windows of 10 beats.

### Acquisition protocol

The intervention was completed in a laboratory (77 meters above sea level) without direct sunlight and with the participant sitting reclined (about 45°) in a dental chair with the forearms supported on armrests. The testing was completed at approximately the same time (starting between 08.00 and 09.00h) and the same room temperature (mean 24.2 °C (± 0.8 °C, SD)) for all participants. Participants wore regular, everyday clothing. If the participants’s fingers were cold, the hands were warmed by manual massaging, and knitted mittens were used. The mean atmospheric pressure was 101.5 ± 1.3 kPa. Hypoxia was introduced by decreasing the fraction of oxygen in the breathing air using a gas blender (Reduced Oxygen Breathing Device 2 (ROBD2, model 6202−1, Environics Inc, CT, USA). Two thermal mass controllers blended normobaric air and nitrogen (N_2_) in volumes resulting in gases containing reduced fractions of oxygen [45]. The ROBD2 is reported to deliver valid fractions of oxygen (O_2_) in the range between 5 and 21% of the total breathing gas content [46]. During the experiment the air flow rate was 50 L min^–1^ and delivered using a standard aircraft breathing mask (MBU-20/P, Gentex Corp, PA, USA) that sealed the mouth and nose from atmospheric air and was individually adjusted for each participant. In addition to data from the internal pulse oximeter, the ROBD2 reported the time, oxygen concentration in the breathing air and equivalent height.

**Fig 2:**
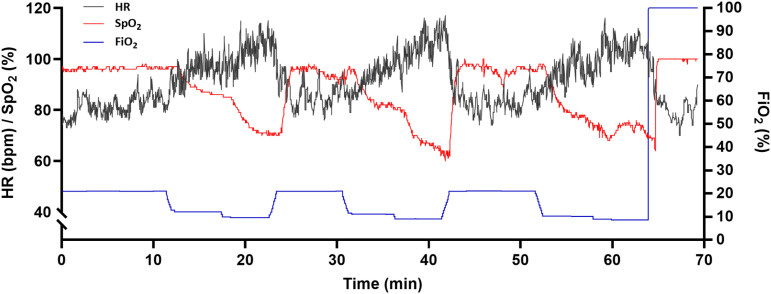
Example of the SpO_2_ and HR response to the hypoxic exposure. The figure displays a typical run from one participant in the study chronologically exposed to FiO_2_s of 20.9, 12, 9.5, 20.9, 11, 9, 20.9, 10 and 8.5%. The X-axis is time (min), left Y-axis heart rate (BPM) and O_2_Hb saturation (SpO_2_, %); right Y-axis, fraction of inspired oxygen (%). Data are readings from one pulse-oximeter (SpO_2_, red line; heart rate, black line) and the FiO_2_ (blue line).

A flowmeter (SFM3300, Sensiron AG, Switzerland) was connected in series with the air hose between the ROBD2 and the breathing mask to measure instantaneous airflow, with an update frequency of 2000 Hz. The instantaneous flow (L s^–1^) was then converted to tidal volume (TV, L), respiration rate (RR, breaths min^–1^) and pulmonary minute ventilation (L min^–1^). To do this, the volume and time data from four consecutive inspirations prior to the time marker were pooled, and from that, the relevant parameters were derived. Pulmonary minute ventilation was also normalized to fat-free mass (ml kg^–1^min^–1^). The participants were exposed to breathing gases with reduced fractions of inspired oxygen (FiO_2_) to introduce stable oxygen saturations in the participants in the following three target intervals: 55–70%, 70.1–85% and 85.1–100% SaO_2_. Since there can be considerable inter-individual variation in oxygen saturation response at a given oxygen fraction [47], a partly pragmatic exposure protocol was designed to account for this variability. The protocol consisted of eight different gas blends (20.9, 12, 11, 10, 9.5, 9, 8.75/8.5, and 8% FiO₂; see Table 4). All participants first inhaled 20.9% oxygen for approximately 10 min, followed by 12% and 9.5% FiO₂ for 3–5 min each, before being reoxygenated with 20.9% FiO₂ for 5–10 min. The subsequent FiO₂ levels were adjusted based on the participant’s oxygen saturation response at the 9.5% FiO₂ step. If SpO₂ remained relatively high (~70–75%), exposure continued with 10% and then 8.5% FiO₂. If SpO₂ was relatively low (~60–65%), exposure proceeded with 11% and then 9% FiO₂. In some cases, the exposures to the intermediate step of 10% FiO_2_ step were excluded. Finally, if the saturation remained above 65% at the last step, a further reduction to 9% or 8% FiO₂ was introduced. Thus, most participants were exposed to 5 (*n* = 8) or 6 (*n* = 7) combinations of breathing gases before reaching the end target saturation interval, while two participants were exposed to 7 combinations. The participants were asked to rest and minimize bodily movement during the intervention. Further to divert the attention from the ongoing measurements the participants watched a nature program on a separate monitor during the intervention. After the final exposure level, the participants were first given 100% oxygen for 5 min and then breathed normal room air without a mask during observation. The examination was also ended if the participant either wanted to abort or if the oxygen saturation fell below 55%. See Table 4 for an overview of the exposure and Fig 2 for the SpO_2_ and HR responses to a typical run.

**Table 4:** The breathing gas exposure protocol.

Air mix. (no.)	Fraction O_2_ (%)	Exposure (min)	Participants (no.)
Ground	20.9	8–10	17
1	12	3–5	17
2	9.5	3–5	17
Ground	20.9	5–10	
3	11	3–5	15
4	9	3–5	15
Ground	20.9	5–10	
5	10	3–5	6
6	8.75/8.5	3–5	5
7	8	3–5	2
Recovery	100	5	17

Overview of the partly pragmatic exposure protocol in the study showing oxygen fractions (%) in the breathing gases, exposure time (min) at the corresponding O_2_ fraction and the resultant number of participants exposed to the given breathing gas combination.

### Data synchronization and missing data

A time marker (TM) was used to synchronize the data and was activated at the start of each blood sampling. Except for the point measurements for the reference SaO_2_ taken from arterial blood sampling, the data from pulse oximeters, ECG, flowmeter and the ROBD2 were sampled continuously and transferred to a separate, standalone PC (Dell, Optiplex 790) using LabView software. These data were subsequently synchronized with the time of the blood samples using the time markers. When stable values of the oxygen saturation were observed, defined as a variation of ≤ 3 pp [48], three to five separate blood samples per breathing gas level were taken successively with 20–30 s interval. In total, 15–21 repeated blood samples from each participant and a total of 304 blood samples were taken. At the TM for each blood sample the simultaneous measured SpO_2_ at that time point for each of the separate pulse oximeters were used in the further analysis. No time averaging was performed for either SpO_2_ or HR. The heart rate measurements used in the examination of validity were taken respectively at 15, 30, 45 and 60 s prior to the first blood sampling/TM at each separate breathing gas step to reduce any impact of the blood sampling procedures. As the participants were exposed to five to seven breathing gases, a total of 20–28 heart rate measurements per participant were taken. In total for all participants, 383 heart rate samples were recorded. For the description of heart rate response to the exposure of air with different oxygen contents, the heart rate synchronized with each blood sample was used. The collected data was reported at 20 Hz and transferred to and further treated in Excel.

Periodically, the ROBD2 software paused the transfer of data to LabView, seemingly at random, and we were not able to locate the origin of this error. Thus, some datapoints from the ROBD2 including the built-in pulse oximeter (Nell1-SR) were missing at random. Because of the minor loss of data relative to the total sampling time (ranging some 80–100 s of data of a total of about 3500 s), the number of missing observations from the ROBD2 at a given time marker was less than one per participant and reaching a total of 13 of 304 observations. All missing data were within ± 2 s of the nearest valid observation. Therefore, this value was imputed as a datapoint for blood oxygen saturation for the ROBD2. Since the data were taken under stable saturation and heart rate conditions, we expect this to have negligeable impact on the results. For the measurements of heart rate in the case of missing data from the ROBD, the measurement time-points were slightly shifted from the planned intervals to the time point of the first valid observation from ROBD2 for all pulse oximeters. As ECG was measured continuously, all heart rate data were therefore always kept synchronized. Because of a temporary connection fault, the ventilation data of one participant are missing. The descriptive resting data were collected and treated as described above. For the data describing the heart rate, hematological and ventilatory responses to the different exposure levels (Table 5), the mean of the 3–5 samples per individual per gas mix were used. All data are collected during the examination, and no data were obtained from sources other than those described.

**Table 5:** Hemoximetry (A), heart rate and ventilation (B) at different levels of FiO_2_.

A	Baseline	12%	11%	9.5%	9%
***N***	17	17	15	17	15
**SaO**_**2**_ **(%)**	97.9 (0.6)^*^	86.0 (2.5)^*^	77.3 (4.0)^*^	70.3 (6.7)^*^	64.1 (8.5)^*^
***p*O**_**2**_ **(kPa)**	12.94 (0.92)^*^	6.62 (0.50)^*^	5.42 (0.41)^*^	4.74 (0.56)^*^	4.35 (0.95)^*^
***p*CO**_**2**_ **(kPa)**	5.02 (0.48)^**^	4.74 (0.33)^**^	4.72 (0.34)	4.48 (0.39)^***^	4.44 (0.38)^***^
**pH** **(–log10[H+])**	7.37 (0.05)^*^	7.39 (0.05)^**^	7.39 (0.05)	7.41 (0.05)^∘^	7.41 (0.05)^∘^
**HCO**_**3**_^**–**^ **(mMol/L)**	21.01 (2.1)	21.04 (2.2)	21.13 (2.4)	21.00 (2.26)	20.74 (2.56)
***p*50 (kPa)**	3.59 (0.19)	3.39 (0.12)^∘∘^	3.42 (0.13)^∘∘^	3.38 (0.11)^∘∘^	3.38 (0.14)^∘∘^
**B**					
***N***	16	16	14	16	14
**HR** **(beats·min**^**–1**^)	67.4 (9.4)^*^	77.4 (10.4)^∘∘∘^	78.1 (10.3)^∘∘∘^	88.9 (11.1)^#^	92.6 (11.5)^#^
**RF** **(insp. min**^**–1**^)	9.5 (3.7)	9.5 (3.8)	9.1 (3.7)	8.8 (2.6)	8.6 (2.6)
**TV (L)**	1.0 (0.6)	1.1 (0.6)	1.1 (0.5)	1.2 (0.5)	1.3 (0.5)
**PMV (L·min**^**–1**^)	7.8 (2.2)	8.6 (1.4) ^##^	8.2 (1.5)^###^	9.6 (1.4)	9.7 (1.6)
**PMV/FFM (ml·kg**^**–1**^**·min**^**–1**^)	127.5 (26.8)	141.6 (17.8)	131.5 (18.3)^###^	158.6 (22.3)^∘^	157.3 (16.2)

Data are given as mean (SD). The individual data for each breathing gas FiO_2_ level are the mean of three to five measurements per participant taken three-five minutes after the initiation of exposure. The effects of oxygen content within each variable were statistically examined using a linear mixed between-within model with the dependent variables and sex as factors. 14 complete pairs of participants were used except for ventilation data (*n* = 13).

Abbreviations. HCO_3_^–^, bicarbonate; HR, heart rate; RF, respiratory frequency; PMV, pulmonary minute ventilation; *p*CO_2_, arterial blood carbon dioxide pressure; *p*O_2_, arterial blood oxygen pressure; p_50_, arterial blood oxygen pressure at 50% Hb oxygen saturation; SaO_2_, functional arterial blood oxygen saturation; TV, tidal volume; SD is standard deviation.

* significantly different from all other values, *p* < 0.001.

** significantly different from all other values except 11% exposure, *p* < 0.05.

*** significantly different from all other values except neighboring exposure levels, *p* < 0.05.

∘ significantly different from all other values except the neighboring 9.5/9% exposure, *p* < 0.05.

∘∘ significantly different from baseline, *p* < 0.05.

∘∘∘ significantly different from all other values except the neighboring 12/11% exposure, *p* < 0.001.

# significantly different from all other values except the neighboring 9.5/9% exposure, *p* < 0.001.

## significantly different from exposure 9.5, *p* < 0.05.

### significantly different from exposures 9.5 and 9%, *p* < 0.001.

### Analysis and statistics

The following measures were used as descriptive variables of the participants in the study: age, sex, body height, body weight, body mass index, absolute and relative fat and muscle mass, forced vital capacity (FVC), forced expiratory volume in 1 s (FEV1), resting blood pressure, oxygen and carbon dioxide pressure (*p*O_2_ and *p*CO_2_), pH, hemoglobin (Hb), hematocrit (Hct), bicarbonate (HCO_3_^–^), oxyhemoglobin (O_2_Hb), deoxyhemoglobin (HHb), carboxyhemoglobin (COHb), methemoglobin (MetHb) and self-reported physical activity. For dichotomous variables, the number and relative values are reported. To statistically examine changes in hemoximetry, heart rate and ventilatory response to the reduced O_2_ exposures and to examine differences in response between the sexes, we used a linear mixed between-within model using the dependent variables and sex as factors. Multiple comparisons were Bonferroni-adjusted. If Mauchly’s test of sphericity was violated, the Greenhouse-Geisser correction was used to adjust for lack of sphericity. The data distributions were evaluated by graphical representations. As uneven number of participants were exposed to the different oxygen concentrations, 14 complete pairs at FiO_2_ levels 20.9, 12, 11, 9.5 and 9% were analyzed for hemoximetry and heart rate and 13 pairs for the ventilation data. The mean of the three to five measurements per participant at each FiO_2_ level was used as data points in the analyses, thus one participant was represented by one data point at each FiO_2_. We found no exposure times sex interactions, and the data were thus pooled for men and women.

To examine the validity of the pulse oximeters at different saturation levels, Bland & Altman’s bias and 95% limits of agreement (LoA), adjusted for repeated measurements [41,42] with exact confidence intervals [41] were computed and reported according to suggested standards [49]. Bias was taken as the value of the pulse oximeter minus the reference value (SaO_2_ or HR), and 95% LoA were taken as the standard deviation of the bias times 1.96 as described by Bland and Altman [40]. To limit the degree of desaturation below 70% SaO_2_, the exposures to reduced FiO_2_ were terminated at a saturation of 55% SaO_2_. This data interval below 70% (15 percentage points wide) was then examined and compared with the two partitioned, equally large intervals above 70% oxygen saturation (70.1–85% and 85.1–100%). The number of participants and observations in the three categories were as follows: 85.1–100%, 17 participants, 100 observations; 70.1–85%, 17 part., 102 obs.; 55–70%, 16 part., 102 obs. Since the measurements of heart rate were taken prior to the blood samples, we therefore used the mean SpO_2_ across the four examined pulse oximeters as indicator for oxygen saturation ranges when partitioning the heat rate data in the three saturation intervals. In the saturation interval 85.1–100% there were 133 observations from 17 participants; 70.1–85%, 147 observations from 16 participants, and in interval 55–70% there were 103 observations from 15 participants. The overall difference (± SD) in oxygen saturation between the mean value of all pulse oximeters compared with that of SaO_2_ was 0.8 pp (2.1), *n* = 304. Since the O_2_ saturation and HR are in principle changing due to the exposure and breathing pattern, immediate replicates were not taken, and thus repeatability of measures was not analyzed in this study. The data were consequently analyzed using the “Method where the true value varies” [42]. The root mean square error (RMSE) has been used as a compound indicator of both accuracy and precision for SpO_2_ and HR of pulse oximeter manufacturers and regulatory authorities [15–17]. We therefore calculated the RMSE (square root of the mean of the squared difference between the pulse oximeter and reference) values of the three saturation intervals (55–70%, 70.1–85% and 85.1–100%) using the equation, $RMSE=\sqrt{\frac{{\Sigma ({SpO}_{2}-{SaO}_{2})}^{2}}{n}}$, where SpO_2_ is the oxygen saturation values of the pulse oximeter, and SaO_2_ is that of the hemoximeter. For heart rate, the values were beats per minute determined by the pulse oximeter and ECG, respectively.

As inference criteria for the Bland-Altman analyses, the maximum allowable difference in oxygen saturation and heart rate between the pulse oximeter and the reference methods was set to ± 6 pp and ± 6 bpm respectively. Those values were based upon the RMSE values reported by the pulse oximeter manufacturers for the interval 70–100% SaO_2_ (3%, [15–17]) and multiplied by 1.96, which corresponds to a 95% LoA of ≈ 6 pp and bpm. The two measurement methods (pulse oximetry and reference measurements) are considered to be in agreement if the higher and lower Limit of Agreement including the 95% CI for these limits, is respectively lower and higher than the maximum allowable difference [50].

To statistically examine the effect of reduced O_2_Hb saturation on the bias and RMSE of O_2_Hb saturation and HR within a pulse oximeter, the SpO_2_ and HR for the three saturation intervals were statistically compared using one-way repeated measures ANOVA with Bonferroni post hoc test adjustment for multiple comparisons. If Mauchly’s test of sphericity was violated, the Greenhouse-Geisser correction was used. For these comparisons, the mean of all observations for each participant within a saturation interval was used, ensuring that each participant was represented by a single observation per saturation interval. For these within-group analyses, there were 16 complete pairs of observations across all three saturation intervals and 15 complete pairs for HR. The data distributions were evaluated by graphical representations. Data are reported as mean ± SD, median (interquartile range) and number. *P*–values less than 0.05 were considered as being statistically significant. Data analysis was completed using either IBM SPSS version 29 or GraphPad Prism version 9.4.1. Adjusted Bland-Altman analyses was completed using the free web-app: https://sec.lumc.nl/method_agreement_analysis/ [41].

## Results

17 healthy participants were examined, and a total of 304 oxygen saturation samples (range 15–21 per participant) and 383 heart rate samples (20–28 measurements per participant) were taken. Baseline data of the participants are given in Table 1 (anthropometrics), Table 2 (hematologic and blood gas values) and Table 3 (hemodynamics and pulmonary function).

### Acute physiological response to reduced oxygen fraction

No statistically significant interactions between exposure times and sex were observed for any physiological response. The mean results of men and women have therefore been pooled (Table 5). SaO_2_ and dissolved O_2_ (*p*O_2_) fell gradually as a function of the reduced oxygen fraction in the breathing air from a mean of 97.9 (0.6)% and 12.9 (0.92) kPa at 20.9% O_2_, to 64.1 (8.5)% and 4.35 (0.95) kPa at 9% O_2_ for SaO_2_ and pO_2_ respectively. There was a large variation between subjects in the saturation response at the lower O_2_ concentrations; while the SD was 0.6 pp at baseline, it rose to 8.5 pp at 9% O_2_.

The *p*CO_2_ decreased from a baseline value of 5.02 (0.48) kPa to 4.72 (0.34) kPa at 11% O_2_ and further to 4.44 (0.38) kPa at 9% O_2_. The mean arterial blood pH was in the normal range across all exposures with a slight increase from 7.37 (0.05) at baseline to 7.41 (0.05) at the 9% FiO_2_ exposure. The concentration of plasma HCO_3_^–^ was stable across all FiO_2_ exposures. *P*_50_ displayed a decrease from 3.59 (0.19) kPa at 20.9 FiO_2_ to 3.39 (0.12) kPa at 12% FiO_2_, after which no further changes were noted. The heart rate increased steadily by reductions in FiO_2_ from a baseline value of 67.4 (9.4)) to 92.6 (11.5) bpm at the 9% FiO_2_. There were no systematic changes in neither respiratory frequency nor tidal volume by changes in FiO_2_, but both the absolute and normalized pulmonary minute ventilation increased at the lower FiO_2_ exposures.

### Agreement between pulse oximeters and reference measurements

#### O_2_ saturation.

**Fig 3:**
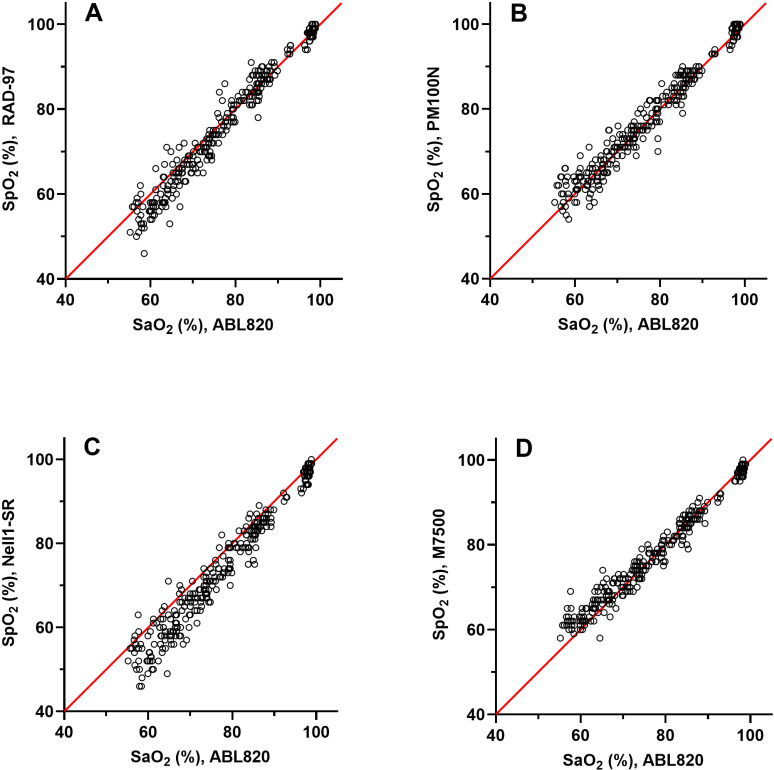
SpO_2_ vs SaO_2_ for the different pulse oximeters. Scatter plots displaying the SpO_2_ of the four pulse-oximeters versus reference SaO_2_ of the hemoximeter (ABL820) for all measurements (*n* = 304). X-axes are SaO_2_ (%), Y-axes are SpO_2_ (%). **A.** RAD-97 vs. ABL820, **B.** PM100N vs. ABL820, **C.** Nell1-SR vs. ABL820, **D.** M7500 vs. ABL820. Line of identity which signifies SpO_2_ = SaO_2_ is marked in red.

**Fig 4:**
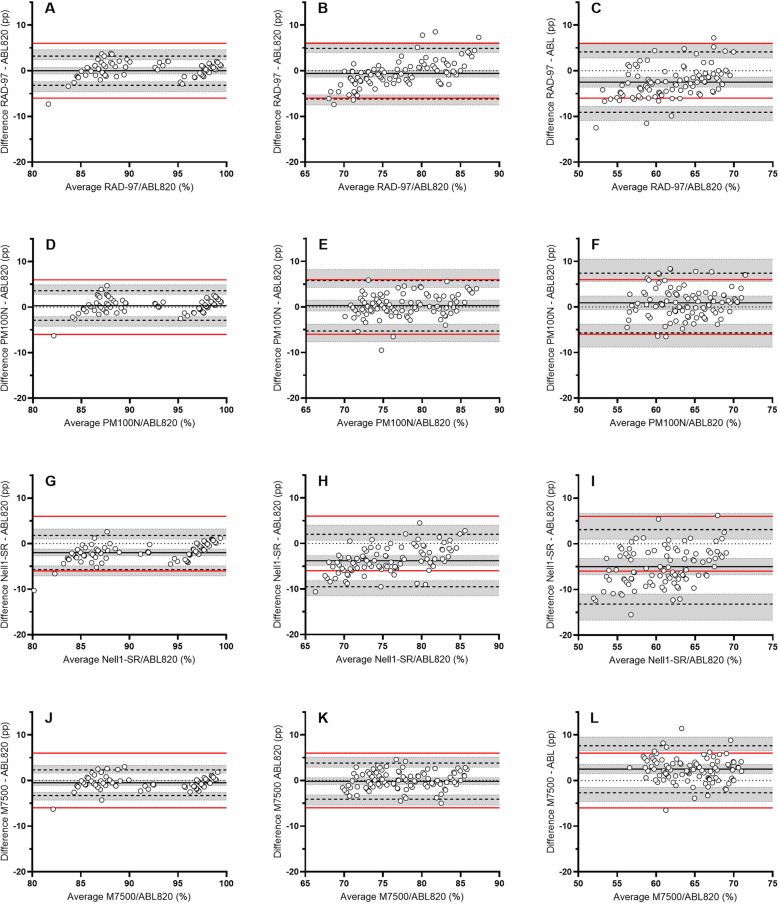
Bland-Altman LoA plots of oxygen saturation for the different pulse oximeters. Bland-Altman plots for Hb oxygen saturation at intervals SaO_2_ 85–100% (A, D, G, J), SaO_2_ 70–85% (B, E, H, K) and SaO_2_ 55–70% (C, F, I, L). Data for RAD-97 are given in A, B, and C; PM100N in D, E and F; Nell1-SR in G, H and I, and M7500 are displayed in J, K and L. X-axis displays the average value of SaO_2_ and SpO_2_.Values on the Y-axis show the difference between SaO_2_ and SpO_2_ and the scaling is equal across plots. Bias (mean difference) is represented by black solid line, limits of agreement (–1.96 SD to + 1.96 SD) are given as black dashed lines, and shaded areas are the 95% CI of the bias and limits of agreement. Zero bias is indicated by dotted black lines, and the red lines signifies the demarcation criteria of 6 pp.

The individual observations from the four different pulse oximeters versus the reference values are shown in Fig 3 for oxygen saturation. The data for Bland-Altman’s bias and LoA, variance and RMSE of oxygen saturation are given in Table 6 and Fig 4. The bias for all stand-alone pulse oximeters were close to zero for saturation levels 85–100 and 70–85%, while for the Nell1-SR PCBA the bias was some –2 and –3.8 pp, respectively. Using the demarcation limits of ± 6 pp, all pulse oximeters except for the Nell1-SR were in agreement with the reference value at oxygen saturation interval 85–100%, while only M7500 displayed LoAs ±95% CI within the demarcation criteria for the interval 70–85%. The pulse oximeters displayed RMSE values less than 3 pp and were thus within the self-reported performance limits for the 85–100% and 70–85% intervals, with one exception. The Nell1-SR displayed 4.7 pp RMSE in the 70–85% interval. In the 55–70% O_2_ saturation interval, the PM100N displayed less than 1 pp of bias, while the offset was –2.5, + 2.5 and –5 pp for RAD-97, M7500 and Nell1-SR, respectively. Also, none of the pulse oximeters displayed LoAs ± 95% CI within the demarcation limits and were not in agreement with the hemoximeter at the lowest levels of O_2_ saturations. Moreover, all pulse oximeters displayed RMSE values greater than 3 pp at this interval.

**Table 6:** Bias and limits of agreement, variance and root mean square error at three oxygen saturation intervals. The pulse oximetry and reference measurements are considered to be in agreement if the higher and lower Limit of Agreement including the 95% CI for these limits, is respectively lower and higher than the maximum allowable difference of ± 6 percentage points.

A.	85–100% O_2_Hb saturation interval			
	RAD-97	PM100N	Nell1-SR	M7500
**Bias (SE)**	0.0 (0.3)	0.3 (0.3)	–2.0 (0.3)	–0.5 (0.3)
**95% CI of the bias**	–0.7 to 0.7	–0.3 to 1.0	–2.7 to –1.2	–1.1 to 0
**SD of the differences (SE)**	1.6 (0.2)	1.6 (0.2)	1.9 (0.2)	1.4 (0.2)
**Limits of agreement (LoA)**	3.2	3.2	3.8	2.8
**Lower LoA (95% CI)**	–3.2 (–4.6 to –2.4)	–2.9 (–4.2 to –2.1)	–5.7 (–7.1 to –4.9)	–3.3 (–4.3 to –2.6)
**Upper LoA (95% CI)**	3.2 (2.4 to 4.6)	3.6 (2.8 to 4.9)	1.8 (1.0 to 3.2)	2.3 (1.6 to 3.3)
**Within-subject variance (SE)**	1.2 (0.2)	1.3 (0.2)	2.2 (0.3)	1.0 (0.2)
**Between-subjects variance (SE)**	1.6 (0.6)	1.4 (0.6)	1.5 (0.7)	1.0 (0.4)
**RMSE**	1.6	1.6	2.6	1.4
**B.**	**70–85% O**_**2**_ **Hb saturation interval**			
	**RAD-97**	**PM100N**	**Nell1-SR**	**M7500**
**Bias (SE)**	–0.6 (0.3)	0.3 (0.6)	–3.8 (0.5)	–0.2 (0.3)
**95% CI of the bias**	–1.4 to 0.1	–0.9 to 1.5	–4.8 to –2.7	–0.9 to 0.6
**SD of the differences (SE)**	2.8 (0.2)	2.8 (0.3)	2.9 (0.3)	2.0 (0.2)
**Limits of agreement (LoA)**	5.6	5.5	5.7	3.9
**Lower LoA (95% CI)**	–6.2 (–7.5 to –5.3)	–5.3 (–7.6 to –3.8)	–9.5 (–11.5 to –8.2)	–4.1 (–5.4 to –3.2)
**Upper LoA (95% CI)**	4.9 (4.0 to 6.2)	5.8 (4.3 to 8.2)	2.0 (0.7 to 4.0)	3.8 (2.9 to 5.1)
**Within-subject variance (SE)**	7.3 (1.1)	3.1 (0.5)	5.1 (0.8)	2.4 (0.4)
**Between-subjects variance (SE)**	0.7 (0.7)	4.9 (1.9)	3.4 (1.6)	1.5 (0.7)
**RMSE**	2.9	2.5	4.7	1.9
**C.**	**55–70% O**_**2**_ **Hb saturation interval**			
	**RAD-97**	**PM100N**	**Nell1-SR**	**M7500**
**Bias (SE)**	–2.5 (0.5)	0.9 (0.7)	–5.0 (0.8)	2.5 (0.5)
**95% CI of the bias**	–3.6 to –1.4	–0.6 to 2.4	–6.8 to –3.2	1.5 to 3.5
**SD of the differences (SE)**	3.4 (0.3)	3.3 (0.4)	4.2 (0.5)	2.6 (0.3)
**Limits of agreement (LoA)**	6.6	6.6	8.2	5.2
**Lower LoA (95% CI)**	–9.1 (–10.9 to –7.8)	–5.7 (–8.8 to –3.8)	–13.2 (–16.7 to –11.0)	–2.7 (–4.6 to –1.5)
**Upper LoA (95% CI)**	4.1 (2.8 to 5.9)	7.4 (5.6 to 10.5)	3.1 (1.0 to 6.6)	7.6 (6.5 to 9.5)
**Within-subject variance (SE)**	8.8 (1.3)	3.8 (0.6)	7.5 (1.1)	4.2 (0.6)
**Between-subjects variance (SE)**	2.4 (1.4)	7.4 (3.0)	9.8 (4.1)	2.8 (1.3)
**RMSE**	4.2	3.4	6.6	3.6

A, interval 85–100% with 100 observations of 17 participants; B, interval 70–85% (102 obs., *n* = 17); C, interval 55–70% (102 obs., *n* = 16). The data are analyzed adjusted for repeated measurements standards [42] and reported according to suggested standards [41,49]. RMSE – Root mean square error.

#### Heart rate.

**Fig 5:**
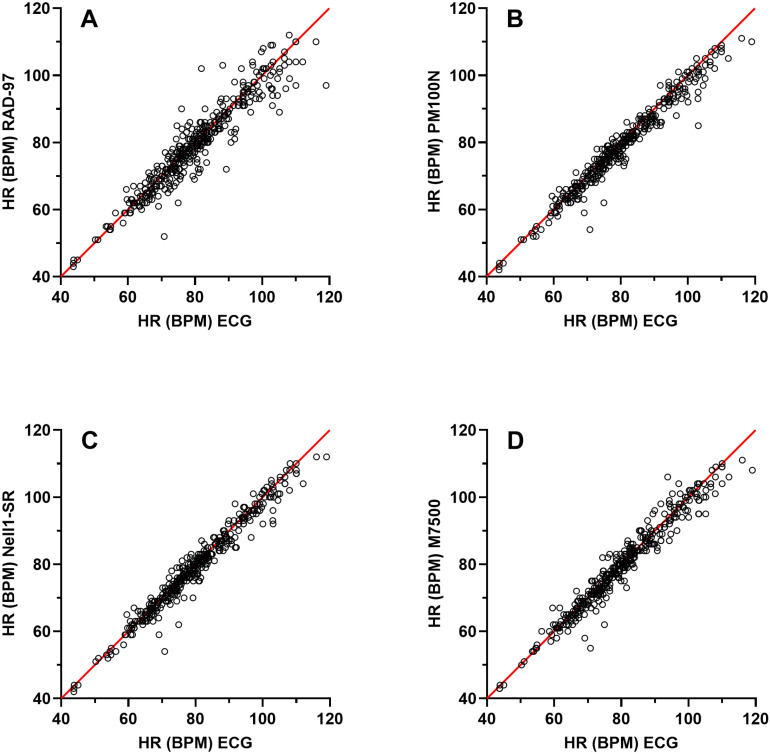
Heart rate by the different pulse oximeters vs heart rate measured by the reference (ECG). Scatter plots displaying the heart rate of the four pulse-oximeters versus reference values from the ECG (*n* = 383). X-axes shows HR by ECG (BPM), Y-axes shows HR by pulse oximeter (BPM). A. RAD-97 vs. ECG, B. PM100N vs. ECG, C. Nell1-SR vs. ECG, D. M7500 vs. ECG. Line of identity signifying that HR pulse oximeter = HR ECG and is marked in red.

**Fig 6:**
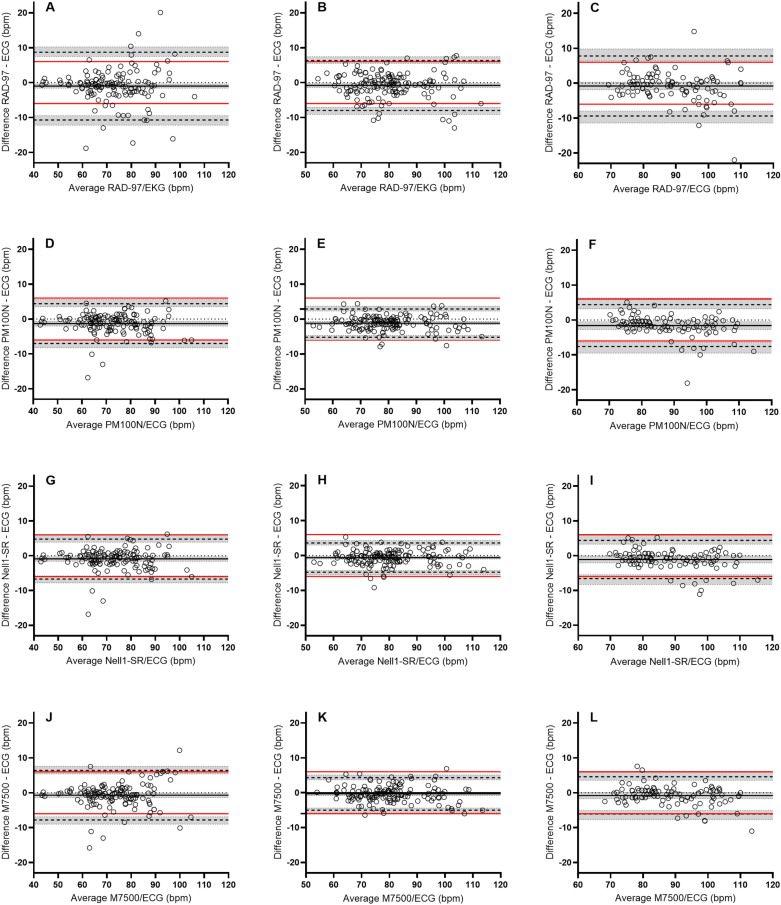
Bland-Altman LoA plots of heart rate for the different pulse oximeters. Bland-Altman plots for heart rate at intervals SaO_2_ 85–100% (A, D, G, J), SaO_2_ 70–85% (B, E, H, K) and SaO_2_ 55–70% (C, F, I, L). Data for RAD-97 are given in A, B, and C; PM100N in D, E and F; Nell1-SR in G, H and I; and M7500 are displayed in J, K and L. X-axis displays the average value of HR from the pulse oximeter and the reference (ECG). Y-axis displays the difference in HR between the ECG and the pulse oximeter with equal scaling across plots. Bias (mean difference) is represented by black solid line, limits of agreement (–1.96 SD to + 1.96 SD) are given in black dashed lines, and shaded area are the 95% CI of the bias and limits of agreement. Zero bias is indicated by dotted black lines and the red lines signifies the demarcation criteria of 6 bpm.

Individual data of heart rate taken from the pulse oximeters versus the ECG reference values are shown in Fig 5. Data for bias and LoA, variance and RMSE of heart rate are given in Table 7 and Fig 6. The mean bias varied between –0.3 and –1.6 bpm for all pulse oximeters across the three saturation intervals. At saturation level 85–100% none of the pulse oximeters displayed LoAs ± 95% CI within the limit criteria (± 6 bpm), and the data were therefore not in agreement with the reference values. Similarly, all pulse oximeters displayed RMSE values larger than 3 bpm at this interval. At the 70–85% saturation, all pulse oximeters except RAD-97 were in agreement with the reference, and they similarly displayed RMSE values less than 3 bpm. At the 55–70% interval none of the pulse oximeters were in agreement with the reference. The RMSE was lower or equal to 3 bpm in the Nell1-SR and M7500 but higher than that value in the RAD-97 and PM100N.

**Table 7:** Bias and limits of agreement, variance and root mean square error for heart rate at the three oxygen saturation intervals. The pulse oximetry and reference measurements are considered to be in agreement if the higher and lower Limit of Agreement including the 95% CI for these limits, is respectively lower and higher than the maximum allowable difference of ± 6 beats per minute.

A.	85–100% O_2_Hb saturation interval			
	RAD-97	PM100N	Nell1-SR	M7500
**Bias (SE)**	–1.0 (0.3)	–1.3 (0.3)	–0.9 (0.3)	–0.7 (0.3)
**95% CI of the bias**	–1.6 to –0.4	–2.0 to –0.6	–1.6 to –0.3	–1.4 to –0.1
**SD of the differences (SE)**	5.0 (0.3)	2.9 (0.2)	2.9 (0.2)	3.6 (0.2)
**Limits of agreement (LoA)**	9.7	5.7	5.8	7.1
**Lower LoA (95% CI)**	–10.7 (–12.2 to –9.5)	–7.0 (–8.2 to –6.1)	–6.7 (–7.8 to –5.8)	–7.8 (–9.0 to –6.9)
**Upper LoA (95% CI)**	8.7 (7.5 to 10.2)	4.4 (3.5 to 5.6)	4.8 (3.9 to 6.0)	6.4 (5.4 to 7.6)
**Within-subject variance (SE)**	26.0 (3.4)	7.8 (1.0)	8.0 (1.1)	13.1 (1.7)
**Between-subjects variance (SE)**	–1.4 (0.8)	0.8 (0.6)	0.6 (0.6)	–0.1 (0.6)
**RMSE**	5.1	3.2	3.1	3.7
**B.**	**70–85% O**_**2**_ **Hb saturation interval**			
	**RAD-97**	**PM100N**	**Nell1-SR**	**M7500**
**Bias (SE)**	–0.8 (0.3)	–1.2 (0.2)	–0.6 (0.2)	–0.3 (0.2)
**95% CI of the bias**	–1.4 to –0.3	–1.6 to –0.7	–1.0 to –0.3	–0.8 to 0.1
**SD of the differences (SE)**	3.7 (0.2)	2.1 (0.1)	2.1 (0.1)	2.3 (0.1)
**Limits of agreement (LoA)**	7.2	4.1	4.2	4.6
**Lower LoA (95% CI)**	–8.0 (–9.1 to –7.1)	–5.2 (–6.0 to –4.7)	–4.8 (–5.5 to –4.3)	–5.0 (–5.8 to –4.4)
**Upper LoA (95% CI)**	6.3 (5.5 to 7.4)	2.9 (2.3 to 3.7)	3.6 (3.0 to 4.3)	4.3 (3.7 to 5.1)
**Within-subject variance (SE)**	13.8 (1.7)	4.1 (0.5)	4.6 (0.6)	5.6 (0.7)
**Between-subjects variance (SE)**	–0.4 (0.4)	0.3 (0.3)	0.0 (0.2)	–0.0 (0.2)
**RMSE**	3.7	2.4	2.2	2.4
**C.**	**55–70% O**_**2**_ **Hb saturation interval**			
	**RAD-97**	**PM100N**	**Nell1-SR**	**M7500**
**Bias (SE)**	–0.8 (0.5)	–1.6 (0.5)	–1.1 (0.4)	–0.8 (0.4)
**95% CI of the bias**	–1.9 to 0.3	–2.7 to –0.5	–2.1 to –0.1	–1.7 to 0.1
**SD of the differences (SE)**	4.4 (0.3)	3.1 (0.3)	2.8 (0.2)	2.7 (0.2)
**Limits of agreement (LoA)**	8.6	6.0	5.5	5.4
**Lower LoA (95% CI)**	–9.4 (–11.4 to –8.0)	–7.6 (–9.5 to –6.4)	–6.6 (–8.3 to –5.5)	–6.1 (–7.7 to –5.1)
**Upper LoA (95% CI)**	7.8 (6.4 to 9.8)	4.4 (3.2 to 6.3)	4.4 (3.3 to 6.1)	4.6 (3.6 to 6.1)
**Within-subject variance (SE)**	18.1 (2.7)	6.9 (1.0)	5.9 (0.9)	6.0 (0.9)
**Between-subjects variance (SE)**	1.3 (1.5)	2.5 (1.3)	1.9 (1.1)	1.5 (0.9)
**RMSE**	4.4	3.4	3.0	2.8

A, interval 85–100% consisting of 133 observations from 17 participants; mean (SD) HR 73.4 (12.1). B, interval 70–85% (147 obs., *n* = 16; mean (SD) HR 80.4 [12]). C, interval 55–70% (103 obs., *n* = 15; mean (SD) HR 88.9 (11.4)). The data have been analyzed adjusted for repeated measurements standards [42] and reported according to suggested standards [41,49]. RMSE, Root mean square error.

### Effect of desaturation on pulse oximeter performance

For three pulse oximeters the mean bias of the SpO_2_ at O_2_Hb saturation interval 55–70% was significantly different compared with the mean bias at the 70–85% and 85–100% intervals (Table 8). For RAD-97 and Nell1-SR, the offset was negatively increased as compared with the 70–85% and 85–100% interval, while for M7500 the O_2_Hb saturation was positively biased. The mean bias of PM100N did not change as a function of desaturation and was close to zero for all saturations examined. The variability of the difference between SpO_2_ and SaO_2_ was also affected by desaturation. The mean RMSE was significantly larger for all pulse oximeters at the 55–70% interval than at the 85–100% interval. For three of the pulse oximeters the variability of difference was also larger at the 70–85% compared with the 55–70% interval. For heart rate, neither bias nor RMSE changed as a function of desaturation for any pulse oximeter (Table 8).

**Table 8:** Effect of O_2_Hb saturation interval levels on bias and root mean quare error.

A.	O_2_Hb saturation				
	O_2_Hb saturation interval (%)	RAD-97	PM100N	Nell1-SR	M7500
**Bias**	**85–100**	–0.1 (1.3)^^^^	0.3 (1.3)	–2.0 (1.4)^^^^	–0.5 (1.1)^^^^^
	**70–85**	–0.7 (1.4)^^^^	0.2 (2.4)	–3.7 (2.2)^‡‡^	–0.2 (1.5)^^^^^
	**55–70**	–2.5 (2.0)	0.9 (2.8)	–5.0 (3.3)	2.5 (1.9)
**RMSE**	**85–100**	1.4 (0.7)^^^^^	1.6 (0.6)^^^	2.4 (1.3)^^^^^	1.3 (0.7)^^^^^
	**70–85**	2.6 (1.1)^^^, ‡‡^	2.4 (1.5)	4.4 (1.8^)^, ‡‡‡^	1.9 (0.7)^^^^, ‡^
	**55–70**	4.0 (1.3)	2.9 (1.7)	6.0 (2.8)	3.3 (1.3)
**B.**	**Heart rate**				
	**O**_**2**_ **Hb saturation interval (%)**	**RAD-97**	**PM100N**	**Nell1-SR**	**M7500**
**Bias**	**85–100**	–0.9 (1.6)	–1.4 (1.5)	–1.1 (1.4)	–0.7 (1.4)
	**70–85**	–0.7 (1.0)	–1.3 (1.1)	–0.8 (1.0)	–0.3 (0.9)
	**55–70**	–0.8 (1.9)	–1.4 (1.6)	–0.9 (1.5)	–0.7 (1.5)
**RMSE**	**85–100**	3.5 (2.5)	2.6 (1.8)	2.5 (1.8)	2.7 (2.2)
	**70–85**	3.1 (1.7)	2.3 (1.1)	2.2 (1.2)	2.2 (0.8)
	**55–70**	3.6 (2.6)	2.6 (1.7)	2.3 (1.5)	2.4 (1.5)

Comparison of bias and root mean squared error (RMSE) of SpO_2_ (A, *n* = 16) and HR (B, *n* = 15) between the separate O_2_Hb saturation intervals within pulse oximeter. Data are mean (SD) percentage points for O_2_Hb saturation and beats per minute for heart rate.

Significantly different from interval 55–70%, ^^^, *p* < 0.05; ^^^^, *p* < 0.01; ^^^^^. *p* < 0.001. Significantly different from interval 85–100%, ^‡^, *p* < 0.05; ^‡‡^, *p* < 0.01; ^‡‡‡^, *p* < 0.001.

## Discussion

As far as we know, this is one of the first independent systematic examinations of pulse oximeter performance at oxygen saturation levels between 55 and 70% in SaO_2_ in healthy participants after the 2000s. We examined a total of 304 parallel samples of O_2_Hb saturation and 383 heart rate samples from 17 healthy female and male participants during profound experimental hypoxia. The pulse oximeters displayed a gradual decrement in SpO_2_ measurement performance with increases in bias and variability by decreased O_2_ saturation. At the 55–70% saturation interval, none of the examined pulse oximeters were in agreement with reference values for O_2_Hb interval using LoA of 6 percentage points (pp) ± 95% CI. Likewise, all RMSE values were above 3 pp for this interval. Desaturation had different effects on the reported heart rates. All pulse oximeters except the RAD-97 agreed with the reference ECG on the measurement of heart rate at the 70–85% saturation interval, but none agreed at the 55–70% nor the 85–100% saturation intervals. However, the observed biases in heart rate were less than 2 bpm, which we deem quite small.

### Acute physiological changes during the hypoxic exposure

Despite data being sampled under stable conditions, we acknowledge that it cannot be ruled out that the physiological responses at a given FiO_2_ level may not have reached steady state due to the relatively brief exposure duration.

The participants displayed normal physiological hemostatic responses to the acute stepwise decreases in breathing air oxygen concentration in order to cope with the reduced oxygen availability [51]. As emphasized above, although the blood samples and heart rate were taken under stable conditions, we cannot assume that the data were recorded under truly steady state conditions for all physiological variables as the primary goal of the study was instrument validation and not physiological responses to hypoxia. During the exposure there was a gradual decrease in *p*O_2_ and SaO_2_ by reductions in FiO_2_. Although the mean *p*O_2_ fell substantially at all FiO_2_ exposures except baseline, there was no clear hypoxic ventilatory response (HVR) in the participants to the exposures. Neither tidal volume nor respiratory frequency increased systematically by decreased FiO_2_. When normalized for fat-free mass, there was a mean increase in pulmonary minute ventilation during the 9.5% FiO_2_ exposure only. This small increase in ventilation was followed by a small, but significant decrease in *p*CO_2_ during exposure. The baseline values for *p*CO_2_ were almost identical to that of reference values at sea level [52], so the moderate increase in ventilation could not be explained by an unusual high baseline value. It is possible that the known large variation in human HVR response [53] and onset of the hypoxic ventilatory decline [54] can have masked a systematic increase in ventilation in the present study. Also, there were minor changes in pH during the short exposures, and these were within the normal values for sea level [52]. The heart rate increased from a mean of 67 bpm at baseline to 93 bpm at the 9% FiO_2_, which is a normal, compensatory response to acute hypoxia [55], mainly as a function of increased sympathetic and reduced parasympathetic activity [56,57].

### Bias and agreement of the pulse oximeters

For O_2_Hb saturation, the three standalone pulse oximeters (Masimo RAD-97, Nellcor PM100N and Nonin M7500) and the manufacturer specific finger sensors performed according to their self-reported accuracy and precision claims under no motion conditions, which generally is less than 3 pp RMSE across the intervals 70–100% (see results for details). For the partitioned intervals 70–85% and 85–100%, all standalone pulse oximeters also displayed RMSE values below 3 pp. In this study the demarcation criterion for considering the pulse oximeters to agree with the reference values were defined to ± 6 pp and ± 6 bpm for SpO_2_ and heart rate, respectively. The ± 6 pp or bpm corresponds to twice the RMSE performance claims of the manufacturers (< 3 pp or bpm) and which is consistent with a 95% LoA of 6 pp or bpm when the bias is zero [15–17]. For the interval 85–100% O_2_Hb saturation, all standalone pulse oximeters displayed biases close to zero, LoAs ± 95% CI within ± 6 pp and were therefore in agreement with the reference values. At the 70–85% O_2_Hb saturation interval, the biases of the three standalone pulse oximeters were also about zero, but at this interval only the Nonin M7500 was in agreement with the reference. The LoAs ± 95% CI of the RAD-97 and PM100N exceeded the 6 pp criteria. The seemingly contradictory conclusions drawn from the RMSE and LoA for the RAD-97 and the PM100N can be explained by a small increase in bias and the inclusion of the 95% CI of the LoA. While the error claims of the manufacturers are based upon the RMSE without any measure of uncertainty, the inclusion of the 95% CI for the LoA causes a widening of the error term that exceeded the 6 pp limits.

The embedded PCBA pulse oximeter Nell1-SR in the ROBD2 gas blender used for the hypoxic exposures was examined with a non-Nellcor finger sensor as it was set up by the gas blender manufacturer. This configuration demonstrated a systematic error with a bias of –2% and LoAs ± 95% CI that exceeded the ± 6 pp agreement limit criteria at the 85–100% SaO_2_ saturation interval. Thus, even under the most well-saturated conditions, this monitor and sensor combination was not in agreement with the reference. At the 70–85% O_2_Hb saturation interval, the bias decreased further to –3.8 pp. For the RMSE, the combination of the Nellcor PCBA and a non-Nellcor sensor displayed values within 3% for the 85–100% interval, but 4.7% at the 70–85% interval, resulting in a pooled RMSE of 3.8% for the 70–100% interval. The failure to operate within reported standard performance through the 70–100% interval for the Nellcor Nell1-SR may likely origin from the use of a non-Nellcor finger sensor. As the PCBA technology and algorithms within the standalone Nellcor PM100N and Nell1-SR are identical according to the manufacturer, this indicates that the increased errors origin from the use of the non-Nellcor sensor. It may be that the non-Nellcor sensor had emission spectra slightly different from that used by Nellcor and which therefore increased the measurement error [58]. Previous studies have shown a large difference in mean bias and variability across the 70–100 O_2_Hb saturation range between different finger sensors attached to the same pulse oximeter monitor [59], which supports this interpretation. Another factor that may have had impact on the errors was that while soft-holster type sensors was used in combination with the three standalone monitors, the non-Nellcor finger sensor was a clip-on type, and it is possible that this sensor is more subjected to noise than the holster type sensors. However, as the performance of the Nellcor PCBA was not control examined using a Nellcor holster type sensor, conclusions about possible effects of the monitor-sensor configuration cannot be drawn from this study.

Under the most severe hypoxic conditions, the 55–70% O_2_Hb saturation interval, none of the examined pulse oximeters were in agreement with the reference (± 6 pp), and neither displayed RMSE values less than 3 pp. Therefore, a clear and general increasing deterioration in performance by decreased saturation was seen for all four pulse oximeters examined. The mean measurement biases were significantly increased at the 55–70% interval compared with the 85–100% interval for three of the examined pulse oximeters. The RAD-97 and Nell1-SR displayed negatively increased biases of respectively –2.5 and –5 pp compared with the reference, that is an underestimation of the oxygen saturation, while the M7500 overestimated the O_2_ saturation by 2.5 pp. The bias of the Nellcor PM100N did not increase significantly at the 55–70% interval (0.9 pp). The mean variability as measured by the RMSE increased further for all pulse oximeters and were significantly larger than that recorded at the 85–100% saturation interval. Reduced oximeter performance by decreased SaO_2_ seems to be a consistent finding across saturation levels and have been shown in both controlled laboratory hypoxia studies in healthy participants [24,60] and in clinical, real-life studies in various patient and age groups [61,62]. At controlled oxygen saturations below 70% the pulse oximeter precision deteriorates even further compared with at higher saturation level [24,63–66]. A multitude of factors are known to affect the measurements of a pulse oximeter, including physical, technical, and physiological factors [9,10,30,67]. The manufacturers use specific algorithms to convert the optical signal of the pulse oximeter to an SpO_2_ value by comparing the signal with a reference SaO_2_ value based on empirical derived calibration curves [9,10,68]. As the calibration involves a set of human participants with a given set of individual characteristics, any errors that are introduced by the empirical calibration itself will therefore limit the inherent accuracy of the pulse oximeter [9,69]. During the calibration, errors cannot only arise from the pulse oximeter–participant interaction, but may also be introduced by the reference hemoximeter [30]. Because of the known difference in performance between reference hemoximeters [70], it is recommended to use two separate hemoximeters in validation studies [30]. In the present study, only one reference hemoximeter was available. A slight difference between the hemoximeter used in this study compared with the hemoximeters used for developing the original calibration curve may therefore have introduced parts of the variation in the measurements. In addition, since the empirical calibration of the pulse oximeters are completed in the 70–100% O_2_Hb saturation range, any estimation of saturation below this range is based on extrapolating from higher saturations [64,69]. It has therefore been advised that any readings below this limit should be taken cautiously [10,69]. The extrapolation to lower saturation values is probably a main cause of the deterioration in performance found at the 55–70% saturation interval in the present study.

Other possible sources of measurement error are related to physical differences between the participants involved in different calibration studies [9]. The differences between a study population and the calibration population in factors known to affect the optical signal by scattering, absorption or reflecting of the photons [71] can therefore have impact on the measurement error across all saturation values. In the present study we have attempted to control other factors known to affect the pulse oximetry measurements through the study inclusion criteria and the implementation of the study protocol. Possible effects of factors like anemia [72], COHb [25] and MetHb [26] were minimal as shown by direct measurements of these parameters showing normal values. The use of nail polish [20,73] was not permitted, and the fingers of all participants were washed using isopropanol before placing the sensors to minimize any external influence on the pulse oximeters light path. A possible limitation in the study is that we did not optically shield the finger sensors from each other during the experiments. It cannot be ruled out that this may have caused some interference between the sensors [74]. However, the sensors were spaced one finger apart (2^nd^ and 4^th^ finger), and three of the sensors were of a soft holster type that is likely more shielded from both receiving and sending interfering light. We therefore assume that a possible interference between the sensors have not been a major problem [74].

The hypoxic exposures were also completed in a “no-motion” approach to limit any effects of movement on the measurements [18,21]. The perfusion of the hands [18,22] were controlled by using standard room temperature in the laboratory, and if the subject felt cold, the hands were warmed, and knitted mittens were used during the examination. All participants were circulatory and respiratory healthy, and none were using vasoactive drugs. A well-known systematic difference in O_2_Hb bias exists between persons with different content in skin chromophores such as melanin [19,60]. In the present study no participants with dark skin pigmentation volunteered, leaving the group homogenous with light skin tone. The lack of variation in skin pigmentation in this study could therefore negatively affect the measurement bias if the calibration curves of the manufacturer was based on empirical data with participants of various degree of pigmentation. However, the variability within the study group may have been less by the same reason.

Pulse oximeters averaging times may have a large impact on the response-time and fluctuations as it functions as a filter of the data in dynamically varying recordings [75]. In the present study all pulse oximeters were adjusted to comparable averaging times of 2–4 s, and the samples were taken under stable saturation conditions. Thus, variations within a sampling period are not likely to have affected the results much. Also, the sampling of blood of the arterial line may take some seconds to complete, and since this blood is thoroughly mixed, the SaO_2_ may be regarded as a sampling time average across the sampling period. The placement of the arterial line in *a. radials* also minimized the time difference between the sampling of the arterial blood and the finger pulse oximeter [30]. For these reasons we believe that any differences in time of sampling had negligible impact on the results. The sensors were placed randomly on the second and forth finger of both hands. Previous studies have not found any differences in oxygen saturation between these fingers [76], and the randomization should provide a statistical control for any potential unknown systematic difference between fingers.

For heart rate the mean bias for all pulse oximeters was within ± 1.6 bpm across all saturation intervals, and it did therefor not display any clear systematic offset. However, the LoA ± 95 CI of all pulse oximeters were outside the demarcation criteria of 6 bpm at both the 85–100% and 55–70% O_2_Hb intervals. The readings were for that reason not in agreement with the ECG reference values. At the 70–85% interval on the other hand, three of the four pulse oximeters were in agreement with the electrocardiogram. No clear relationship between pulse oximeter performance and saturation level for the measurement of heart rate were therefor seen. Moreover, no statistical differences in bias nor variability were found between the saturation intervals. These results are similar to what others have found, i.e., pulse oximetry display little bias, but relatively large measurement variability as compared with ECG measurement of heart rate [77,78].

In the present study, we show that under controlled laboratory conditions, the performance of the examined pulse oximeters worsen by reduced oxygen saturations, in that the measurement difference range between the pulse oximeters and the reference gradually increase as saturation decrease. At the lowest saturation interval, the three standalone pulse oximeters display ± LoAs of 5–7 percentage points, indicating that for a measured pulse oximeter value displaying 55% saturation with zero bias, the true, unknown SaO_2_ could be some 49–61% for a given individual. In a non-laboratory situation, for example during high altitudes where there is less control of the factors known to affect the validity of the oximeters, it is expected that the differences between the measurement difference between a pulse oximeter and the reference would likely increase and thus the performance of the pulse oximeters to worsen. Indeed, large measurement differences between pulse oximetry and arterial blood gas have been reported previously during Mount Everest expedition [79].

## Conclusions

For a healthy population while controlling for other factors that can affect pulse oximeter performance, the examined standalone pulse oximeters return reasonable performance for measuring O_2_ saturation at the 70–100% saturation interval on average. However, for a single individual, the measurements can vary by several percentage points compared to the true values. At severe arterial desaturation (<70% SaO_2_) none of the examined pulse oximeters were in agreement with the reference. The measurement of heart rate agreed with the electrocardiogram at the saturation interval 70–85% but not at the 55–70% or 85–100% saturation intervals.

## References

[1] Wilson MH, Newman S, Imray CH. The cerebral effects of ascent to high altitudes. Lancet Neurol. 2009;8(2):175–91. doi: 10.1016/S1474-4422(09)70014-6 19161909

[2] McMorris T, Hale BJ, Barwood M, Costello J, Corbett J. Effect of acute hypoxia on cognition: A systematic review and meta-regression analysis. Neurosci Biobehav Rev. 2017;74(Pt A):225–32. doi: 10.1016/j.neubiorev.2017.01.019 28111267

[3] Ottestad W, Hansen TA, Pradhan G, Stepanek J, Høiseth LØ, Kåsin JI. Acute hypoxia in a simulated high-altitude airdrop scenario due to oxygen system failure. J Appl Physiol (1985). 2017;123(6):1443–50. doi: 10.1152/japplphysiol.00169.2017 28839003

[4] ICAO. Manual of the ICAO standard atmosphere. Extended to 80 kilometres (262 500 feet). Third edition ed. Montreal, Canada: International Civil Aviation Organization; 1993.

[5] Peacock AJ. ABC of oxygen: oxygen at high altitude. BMJ. 1998;317(7165):1063–6. doi: 10.1136/bmj.317.7165.1063 9774298 PMC1114067

[6] Rowling SC, Fløjstrup M, Henriksen DP, Viberg B, Hallenberg C, Lindholt JS, et al. Arterial blood gas analysis: as safe as we think? A multicentre historical cohort study. ERJ Open Res. 2022;8(1):00535–2021. doi: 10.1183/23120541.00535-2021 35237684 PMC8883174

[7] Aoyagi T. Pulse oximetry: its invention, theory, and future. J Anesth. 2003;17(4):259–66. doi: 10.1007/s00540-003-0192-6 14625714

[8] Miyasaka K, Shelley K, Takahashi S, Kubota H, Ito K, Yoshiya I, et al. Tribute to Dr. Takuo Aoyagi, inventor of pulse oximetry. J Anesth. 2021;35(5):671–709. doi: 10.1007/s00540-021-02967-z 34338865 PMC8327306

[9] Nitzan M, Romem A, Koppel R. Pulse oximetry: fundamentals and technology update. Med Devices (Auckl). 2014;7:231–9. 25031547 10.2147/MDER.S47319 PMC4099100

[10] Chan ED, Chan MM, Chan MM. Pulse oximetry: understanding its basic principles facilitates appreciation of its limitations. Respir Med. 2013;107(6):789–99. doi: 10.1016/j.rmed.2013.02.004 23490227

[11] Ascha M, Bhattacharyya A, Ramos JA, Tonelli AR. Pulse oximetry and arterial oxygen saturation during cardiopulmonary exercise testing. Med Sci Sports Exerc. 2018;50(10):1992–7. doi: 10.1249/MSS.0000000000001658 29771822 PMC6138536

[12] Durand F, Raberin A. Exercise-induced hypoxemia in endurance athletes: consequences for altitude exposure. Front Sports Act Living. 2021;3:663674. doi: 10.3389/fspor.2021.663674 33981992 PMC8107360

[13] Baumgartner RW, Keller S, Regard M, Bärtsch P. Flunarizine in prevention of headache, ataxia, and memory deficits during decompression to 4559 m. High Alt Med Biol. 2003;4(3):333–9. doi: 10.1089/152702903769192287 14561238

[14] Dünnwald T, Kienast R, Niederseer D, Burtscher M. The use of pulse oximetry in the assessment of acclimatization to high altitude. Sensors (Basel). 2021;21(4):1263. doi: 10.3390/s21041263 33578839 PMC7916608

[15] Masimo. Rad-97® Pulse CO-Oximeter. Operator’s Manual. Irvine, CA, USA: Masimo Corporation; 2020.

[16] Covidien. Bedside SpO2 Patient Monitoring System. Operators;s Manual. Mansfield, MA, USA: Covidien; 2018.

[17] Nonin. Operator’s Manual. Model 7500FO. Pulse Oximeter. North Plymouth, Minnesota, USA: Nonin Medical, Inc.; 2019.

[18] Louie A, Feiner JR, Bickler PE, Rhodes L, Bernstein M, Lucero J. Four types of pulse oximeters accurately detect hypoxia during low perfusion and motion. Anesthesiology. 2018;128(3):520–30. doi: 10.1097/ALN.0000000000002002 29200008

[19] Al-Halawani R, Charlton PH, Qassem M, Kyriacou PA. A review of the effect of skin pigmentation on pulse oximeter accuracy. Physiol Meas. 2023;44(5):05TR01. doi: 10.1088/1361-6579/acd51a 37172609 PMC10391744

[20] Aggarwal AN, Agarwal R, Dhooria S, Prasad KT, Sehgal IS, Muthu V. Impact of fingernail polish on pulse oximetry measurements: a systematic review. Respir Care. 2023;68(9):1271–80. doi: 10.4187/respcare.10399 37185113 PMC10468177

[21] Giuliano KK, Bilkovski RN, Beard J, Lamminmäki S. Comparative analysis of signal accuracy of three SpO2 monitors during motion and low perfusion conditions. J Clin Monit Comput. 2023;37(6):1451–61. doi: 10.1007/s10877-023-01029-x 37266709 PMC10651546

[22] Poorzargar K, Pham C, Ariaratnam J, Lee K, Parotto M, Englesakis M, et al. Accuracy of pulse oximeters in measuring oxygen saturation in patients with poor peripheral perfusion: a systematic review. J Clin Monit Comput. 2022;36(4):961–73. doi: 10.1007/s10877-021-00797-8 35119597

[23] Seifi S, Khatony A, Moradi G, Abdi A, Najafi F. Accuracy of pulse oximetry in detection of oxygen saturation in patients admitted to the intensive care unit of heart surgery: comparison of finger, toe, forehead and earlobe probes. BMC Nurs. 2018;17:15. doi: 10.1186/s12912-018-0283-1 29692684 PMC5905124

[24] Feiner JR, Severinghaus JW, Bickler PE. Dark skin decreases the accuracy of pulse oximeters at low oxygen saturation: the effects of oximeter probe type and gender. Anesth Analg. 2007;105(6 Suppl):S18–23. doi: 10.1213/01.ane.0000285988.35174.d9 18048893

[25] Hampson NB. Pulse oximetry in severe carbon monoxide poisoning. Chest. 1998;114(4):1036–41. doi: 10.1378/chest.114.4.1036 9792574

[26] Feiner JR, Bickler PE, Mannheimer PD. Accuracy of methemoglobin detection by pulse CO-oximetry during hypoxia. Anesth Analg. 2010;111(1):143–8. doi: 10.1213/ANE.0b013e3181c91bb6 20007731

[27] Feiner JR, Rollins MD, Sall JW, Eilers H, Au P, Bickler PE. Accuracy of carboxyhemoglobin detection by pulse CO-oximetry during hypoxemia. Anesth Analg. 2013;117(4):847–58. doi: 10.1213/ANE.0b013e31828610a0 23477959 PMC4476500

[28] Singh AK, Sahi MS, Mahawar B, Rajpurohit S. Comparative evaluation of accuracy of pulse oximeters and factors affecting their performance in a tertiary intensive care unit. J Clin Diagn Res. 2017;11(6):OC05–8. doi: 10.7860/JCDR/2017/24640.9961 28764215 PMC5535407

[29] Wilson BJ, Cowan HJ, Lord JA, Zuege DJ, Zygun DA. The accuracy of pulse oximetry in emergency department patients with severe sepsis and septic shock: a retrospective cohort study. BMC Emerg Med. 2010;10:9. doi: 10.1186/1471-227X-10-9 20444248 PMC2876142

[30] Batchelder PB, Raley DM. Maximizing the laboratory setting for testing devices and understanding statistical output in pulse oximetry. Anesth Analg. 2007;105(6 Suppl):S85–94. doi: 10.1213/01.ane.0000268495.35207.ab 18048904

[31] Mtetwa TK, Zeiler GE, Laubscher L, Pfitzer S, Meyer LCR. Evaluation of the reliability of pulse oximetry, at different attachment sites, to detect hypoxaemia in immobilized impala (Aepyceros melampus). Vet Anaesth Analg. 2020;47(3):323–33. doi: 10.1016/j.vaa.2019.08.051 32278648

[32] Burns PM, Driessen B, Boston R, Gunther RA. Accuracy of a third (Dolphin Voyager) versus first generation pulse oximeter (Nellcor N-180) in predicting arterial oxygen saturation and pulse rate in the anesthetized dog. Vet Anaesth Analg. 2006;33(5):281–95. doi: 10.1111/j.1467-2995.2005.00271.x 16916350

[33] Bazurto Zapata MA, Dueñas Meza E, Jaramillo C, Maldonado Gomez D, Torres Duque C. Sleep apnea and oxygen saturation in adults at 2640 m above sea level. Sleep Sci. 2014;7(2):103–6. 26483911 10.1016/j.slsci.2014.09.003 PMC4521663

[34] Singh B, Cable GG, Hampson GV, Pascoe GD, Corbett M, Smith A. Hypoxia awareness training for aircrew: a comparison of two techniques. Aviat Space Environ Med. 2010;81(9):857–63. doi: 10.3357/asem.2640.2010 20824992

[35] Wen D, Tu L, Wang G, Gu Z, Shi W, Liu X. Psychophysiological responses of pilots in hypoxia training at 7000 and 7500 m. Aerospace Med Human Perf. 2020;91(10):785–9. 10.3357/AMHP.5634.2020 33187564

[36] Tannheimer M, van der Spek R, Brenner F, Lechner R, Steinacker JM, Treff G. Oxygen saturation increases over the course of the night in mountaineers at high altitude (3050-6354 m). J Travel Med. 2017;24(5). 10.1093/jtm/tax041 28931132

[37] Sutton JR, Reeves JT, Wagner PD, Groves BM, Cymerman A, Malconian MK, et al. Operation Everest II: oxygen transport during exercise at extreme simulated altitude. J Appl Physiol (1985). 1988;64(4):1309–21. doi: 10.1152/jappl.1988.64.4.1309 3132445

[38] Malle C, Bourrilhon C, Quinette P, Laisney M, Eustache F, Piérard C. Physiological and cognitive effects of acute normobaric hypoxia and modulations from oxygen breathing. Aerosp Med Hum Perform. 2016;87(1):3–12. doi: 10.3357/AMHP.4335.2016 26735227

[39] Collins J-A, Rudenski A, Gibson J, Howard L, O’Driscoll R. Relating oxygen partial pressure, saturation and content: the haemoglobin-oxygen dissociation curve. Breathe (Sheff). 2015;11(3):194–201. doi: 10.1183/20734735.001415 26632351 PMC4666443

[40] Bland JM, Altman DG. Statistical methods for assessing agreement between two methods of clinical measurement. Lancet. 1986;1(8476):307–10. 2868172

[41] Olofsen E, Dahan A, Borsboom G, Drummond G. Improvements in the application and reporting of advanced Bland-Altman methods of comparison. J Clin Monit Comput. 2015;29(1):127–39. doi: 10.1007/s10877-014-9577-3 24806333

[42] Bland JM, Altman DG. Agreement between methods of measurement with multiple observations per individual. J Biopharm Stat. 2007;17(4):571–82. doi: 10.1080/10543400701329422 17613642

[43] Lu MJ, Zhong WH, Liu YX, Miao HZ, Li YC, Ji MH. Sample size for assessing agreement between two methods of measurement by Bland-Altman method. Int J Biostat. 2016;12(2). 10.1515/ijb-2015-0039 27838682

[44] Kurtze N, Rangul V, Hustvedt B-E. Reliability and validity of the international physical activity questionnaire in the Nord-Trøndelag health study (HUNT) population of men. BMC Med Res Methodol. 2008;8:63. doi: 10.1186/1471-2288-8-63 18844976 PMC2577099

[45] Environics. ROBD2 - Reduced Oxygen Breathing Device 2. Programming and Technical Guide. Tolland, CT, USA; 2013.

[46] Still DL, Temme LA. An independent, objective calibration check for the reduced oxygen breathing device. Aviat Space Environ Med. 2012;83(9):902–8. doi: 10.3357/asem.3046.2012 22946356

[47] Näslund E, Lindberg L-G, Lund I, Näslund-Koch L, Larsson A, Frithiof R. Measuring arterial oxygen saturation from an intraosseous photoplethysmographic signal derived from the sternum. J Clin Monit Comput. 2020;34(1):55–62. doi: 10.1007/s10877-019-00289-w 30805761 PMC6946764

[48] Morey TE, Rice MJ, Vasilopoulos T, Dennis DM, Melker RJ. Feasibility and accuracy of nasal alar pulse oximetry. Br J Anaesth. 2014;112(6):1109–14. doi: 10.1093/bja/aeu095 24736392

[49] Abu-Arafeh A, Jordan H, Drummond G. Reporting of method comparison studies: a review of advice, an assessment of current practice, and specific suggestions for future reports. Br J Anaesth. 2016;117(5):569–75. doi: 10.1093/bja/aew320 27799171

[50] Stöckl D, Rodríguez Cabaleiro D, Van Uytfanghe K, Thienpont LM. Interpreting method comparison studies by use of the bland-altman plot: reflecting the importance of sample size by incorporating confidence limits and predefined error limits in the graphic. Clin Chem. 2004;50(11):2216–8. 15502104 10.1373/clinchem.2004.036095

[51] Winslow RM. The role of hemoglobin oxygen affinity in oxygen transport at high altitude. Respir Physiol Neurobiol. 2007;158(2–3):121–7. doi: 10.1016/j.resp.2007.03.011 17449336

[52] Crapo RO, Jensen RL, Hegewald M, Tashkin DP. Arterial blood gas reference values for sea level and an altitude of 1,400 meters. Am J Respir Crit Care Med. 1999;160(5 Pt 1):1525–31. doi: 10.1164/ajrccm.160.5.9806006 10556115

[53] Weil JV. Variation in human ventilatory control-genetic influence on the hypoxic ventilatory response. Respir Physiol Neurobiol. 2003;135(2–3):239–46. doi: 10.1016/s1569-9048(03)00048-x 12809623

[54] Teppema LJ, Dahan A. The ventilatory response to hypoxia in mammals: mechanisms, measurement, and analysis. Physiol Rev. 2010;90(2):675–754. 20393196 10.1152/physrev.00012.2009

[55] Vogel JA, Harris CW. Cardiopulmonary responses of resting man during early exposure to high altitude. J Appl Physiol. 1967;22(6):1124–8. 5338456 10.1152/jappl.1967.22.6.1124

[56] Joyce W, Wang T. Regulation of heart rate in vertebrates during hypoxia: A comparative overview. Acta Physiol (Oxf). 2022;234(3):e13779. doi: 10.1111/apha.13779 34995393

[57] Kane AD, Kothmann E, Giussani DA. Detection and response to acute systemic hypoxia. BJA Educ. 2020;20(2):58–64. doi: 10.1016/j.bjae.2019.10.004 33456931 PMC7807956

[58] Milner QJW, Mathews GR. An assessment of the accuracy of pulse oximeters. Anaesthesia. 2012;67(4):396–401. doi: 10.1111/j.1365-2044.2011.07021.x 22324874

[59] Choi B-M, Kang BJ, Yun H-Y, Jeon B, Bang J-Y, Noh G-J. Performance of the MP570T pulse oximeter in volunteers participating in the controlled desaturation study: a comparison of seven probes. Anesth Pain Med (Seoul). 2020;15(3):371–7. doi: 10.17085/apm.20028 33329838 PMC7713831

[60] Leeb G, Auchus I, Law T, Bickler P, Feiner J, Hashi S, et al. The performance of 11 fingertip pulse oximeters during hypoxemia in healthy human participants with varied, quantified skin pigment. EBioMedicine. 2024;102:105051. doi: 10.1016/j.ebiom.2024.105051 38458110 PMC10943300

[61] Harris BU, Char DS, Feinstein JA, Verma A, Shiboski SC, Ramamoorthy C. Accuracy of pulse oximeters intended for hypoxemic pediatric patients. Pediatr Crit Care Med. 2016;17(4):315–20. doi: 10.1097/PCC.0000000000000660 26914626

[62] Ruppel H, Makeneni S, Faerber JA, Lane-Fall MB, Foglia EE, O’Byrne ML, et al. Evaluating the accuracy of pulse oximetry in children according to race. JAMA Pediatr. 2023;177(5):540–3. doi: 10.1001/jamapediatrics.2023.0071 36939727 PMC10028535

[63] Hannhart B, Haberer JP, Saunier C, Laxenaire MC. Accuracy and precision of fourteen pulse oximeters. Eur Respir J. 1991;4(1):115–9. doi: 10.1183/09031936.93.04010115 2026231

[64] Severinghaus JW, Naifeh KH. Accuracy of response of six pulse oximeters to profound hypoxia. Anesthesiology. 1987;67(4):551–8. doi: 10.1097/00000542-198710000-00017 3662082

[65] Severinghaus JW, Naifeh KH, Koh SO. Errors in 14 pulse oximeters during profound hypoxia. J Clin Monit. 1989;5(2):72–81. 2723709 10.1007/BF01617877

[66] Bickler PE, Feiner JR, Severinghaus JW. Effects of skin pigmentation on pulse oximeter accuracy at low saturation. Anesthesiology. 2005;102(4):715–9. doi: 10.1097/00000542-200504000-00004 15791098

[67] Tekin K, Karadogan M, Gunaydin S, Kismet K. Everything about pulse oximetry-part 1: history, principles, advantages, limitations, inaccuracies, cost analysis, the level of knowledge about pulse oximeter among clinicians, and pulse oximetry versus tissue oximetry. J Intensive Care Med. 2023;38(9):775–84. doi: 10.1177/08850666231185752 37437083

[68] Nitzan M, Nitzan I, Arieli Y. The various oximetric techniques used for the evaluation of blood oxygenation. Sensors (Basel). 2020;20(17):4844. doi: 10.3390/s20174844 32867184 PMC7506757

[69] Sinex JE. Pulse oximetry: principles and limitations. Am J Emerg Med. 1999;17(1):59–67. doi: 10.1016/s0735-6757(99)90019-0 9928703

[70] Gehring H, Duembgen L, Peterlein M, Hagelberg S, Dibbelt L. Hemoximetry as the “gold standard”? Error assessment based on differences among identical blood gas analyzer devices of five manufacturers. Anesth Analg. 2007;105(6 Suppl):S24–30. doi: 10.1213/01.ane.0000268713.58174.cc 18048894

[71] Lister T, Wright PA, Chappell PH. Optical properties of human skin. J Biomed Opt. 2012;17(9):90901–1. doi: 10.1117/1.JBO.17.9.090901 23085902

[72] Severinghaus JW, Koh SO. Effect of anemia on pulse oximeter accuracy at low saturation. J Clin Monit. 1990;6(2):85–8. 2352007 10.1007/BF02828282

[73] Hinkelbein J, Genzwuerker HV, Sogl R, Fiedler F. Effect of nail polish on oxygen saturation determined by pulse oximetry in critically ill patients. Resuscitation. 2007;72(1):82–91. doi: 10.1016/j.resuscitation.2006.06.024 17098347

[74] Kyriacou PA. Optical crosstalk and other forms of light interference in pulse oximeter comparison studies. J Clin Monit Comput. 2023;37(6):1481–8. doi: 10.1007/s10877-023-01060-y 37610524 PMC10651698

[75] McClure C, Jang SY, Fairchild K. Alarms, oxygen saturations, and SpO2 averaging time in the NICU. J Neonatal Perinatal Med. 2016;9(4):357–62. doi: 10.3233/NPM-16162 27834782 PMC5684874

[76] Basaranoglu G, Bakan M, Umutoglu T, Zengin SU, Idin K, Salihoglu Z. Comparison of SpO2 values from different fingers of the hands. Springerplus. 2015;4:561. doi: 10.1186/s40064-015-1360-5 26543696 PMC4627972

[77] Opio MO, Kellett J, Kitovu Hospital Study Group. How well are pulses measured? Practice-based evidence from an observational study of acutely ill medical patients during hospital admission. Am J Med. 2017;130(7):863.e13-863.e16. doi: 10.1016/j.amjmed.2017.01.033 28235461

[78] Dawson JA, Saraswat A, Simionato L, Thio M, Kamlin COF, Owen LS, et al. Comparison of heart rate and oxygen saturation measurements from Masimo and Nellcor pulse oximeters in newly born term infants. Acta Paediatr. 2013;102(10):955–60. doi: 10.1111/apa.12329 23800004

[79] Prosperi P, Verratti V, Taverna A, Rua R, Bonan S, Rapacchiale G, et al. Ventilatory function and oxygen delivery at high altitude in the Himalayas. Respir Physiol Neurobiol. 2023;314:104086. doi: 10.1016/j.resp.2023.104086 37257573

